# Development of a multifactorial prediction model for commute mode choice in 10 983 Finnish public sector employees: a cross-sectional study

**DOI:** 10.1136/bmjopen-2023-080276

**Published:** 2024-10-16

**Authors:** Anna Makkonen, Kia Gluschkoff, Jaakko Airaksinen, Jaana I Halonen, Paula Salo, Jenni Ervasti

**Affiliations:** 1 Department of Psychology and Speech-Language Pathology, University of Turku, Turku, Finland; 2 Finnish Institute of Occupational Health, Helsinki, Finland; 3 Department of Public Health, Finnish Institute for Health and Welfare, Helsinki, Finland

**Keywords:** statistics & research methods, epidemiology, public health

## Abstract

**Abstract:**

**Objective:**

The objective of this study is to examine the feasibility of using survey data to identify factors that predict commute mode choice.

**Design:**

The study design is cross-sectional.

**Setting:**

Survey data from the Finnish Public Sector study (2020) were used.

**Participants:**

42 574 public sector employees, of whom 10 983 were selected for the final sample. These included employees with 5 km or less commuting distances and those working full-time onsite or partly remotely. The mean age was 46 (SD 11) years, and 84% were women.

**Primary outcomes:**

Commute by (1) bike or foot (an active mode) during summer and winter weather and (2) by car (a passive mode) during summer and winter weather.

**Methods:**

Using logistic Lasso (least-absolute-shrinkage-and-selection-operator) regression, we developed and tested a prediction model for short commutes of 5 km or less to identify the characteristics of employees most likely to commute actively during summer and winter weather and passively during summer and winter weather.

**Results:**

All models had a good predictive ability with a C-index of 0.82, 0.77, 0.72 and 0.71. Cycling and walking during summer weather were predicted by shorter commutes, higher physical activity, lower body mass index (BMI), female sex and higher team psychological safety. Predictors of cycling and walking during winter weather were shorter commute length, higher physical activity, lower BMI and higher age. Commuting by car during summer weather was predicted by longer journey length, higher BMI, lower physical activity, male sex and having children 7–18 years old living at home. Predictors of driving during winter weather were almost identical, but the male sex was replaced by having a spouse.

**Conclusions:**

We identified the correlates of active and passive commute choice in different weather conditions with eight variables. This information can be used to develop and target interventions to promote sustainable and healthy commuting modes.

**STRENGTHS AND LIMITATIONS OF THIS STUDY**

- The strength of this study was the large sample of employees with a high response rate.
- The data used in this study are from a cohort of public sector employees, and the number of women in the sample was predominant.
- No information was available on the commuting environment other than commute distance, for example, information on cycle routes or parking places at the workplace was lacking.
- The cross-sectional design precludes causal conclusions.

## Introduction

 Active commute modes, such as cycling or walking, are simple, cost-effective and sustainable alternatives to driving and are increasingly recognised as an essential source of physical activity.^1^ Cycling has become a crucial mode of transport in the context of policy objectives to mitigate climate change caused by carbon dioxide emissions from transport. Despite the various benefits, active commuting remains low in many countries, and private car use still accounts for a significant proportion of commutes.^2^

Finland is committed to reducing domestic transport emissions by at least 50% compared with 2005 levels by 2030 and carbon neutrality by 2035. The aim is to increase the number of walking and cycling journeys by 30% from 2018 to 2030.^3^ As a comparison, the leading example for commute cycling, Copenhagen, strives for carbon neutrality by 2025, aiming for a 50% modal share for commute cycling and a 75% combined share of walking, cycling and public transport for all trip purposes.^4^ However, at the EU level (including the UK), a large study found no indications that cycling would have been substituted for travel on foot, by public transport, or by car from the 1990s to date.^5^ Instead, the distances travelled by car increased by about 10% during the study period.^5^

In Finland, nearly 50% of the short 1–3 km trips are made by private car, and only 11% by bicycle.^6^ For journeys of 5–10 km, the proportion for cycling drops to 6%. While the average commuting distance in Finland in 2021 was 6 km, 73% of the trips were made by private car, and only 11% and 8% of commuting trips were made by bicycle or by foot.^6^ For commuting distances of 1–10 km, cycling would be a competitive mode of transportation in terms of speed compared with walking, public transportation and private cars, particularly in city centres.

Previous studies have shown that commute mode decisions are based on many different factors of commuters' personal and journey characteristics and active and passive commuting may be predicted by different factors. In general, commuting length and duration seem to be fundamental determinants of commute mode choice.^7^,^10^ Driving is often considered the fastest way to commute. Men commute by car more often than women in several parts of the world, including the Nordic countries like Finland and Sweden.^6 9 11 12^

Sociodemographic factors explain the differences between cyclists and non-cyclists. Particularly in low or emerging cycling cultures like the USA, the UK or Australia, men commute by bicycle more than women. In contrast, in high-cycling countries like the Netherlands, Denmark and Germany, sex and age distribution is more balanced.^13 14^ A Danish study indicates that cyclists are more likely to be men when commuting distance increases (>5 km).^8^

Regarding education and income, the connection to commuting mode choice is often present but generally weaker than other explanatory factors. Higher education and income are associated with active commuting and public transportation use, and, in turn, are associated with a lower likelihood of using a car.^9 11 15 16^ In contrast, lower education is usually associated with commuting by car.^7 17 18^ According to a review, those choosing a carless, proenvironmental lifestyle are often more educated, employed and have higher income.^16^ Low-income usually associates with a higher probability of choosing active commuting modes or public transportation,^9 18^ though this choice is often driven by necessity rather than environmental consciousness. The propensity for households to select sustainable travel options over cars diminishes as car ownership rises.^6 19 20^ Notably, in high-cycling countries like Germany, car ownership levels do not inhibit cycling as much as in Finland.^14^

Although studies on commuting mode choice have steadily increased in the recent decade,^14^ there is still a gap in knowledge on how factors other than socioeconomic characteristics, such as health or psychosocial work environment, contribute to commute mode choice. Variables related to work, teamwork, cooperation and leadership in the organisation are essential, as the behaviour of other people influences the behaviour of an individual. In a tight working community, norms can have a stronger influence than in work communities where coworker relationships are not as close. Identifying individuals likely to commute in a particular way could help in designing effective interventions that encourage switching to a more sustainable and healthier commute mode. We examined the feasibility of using survey data on health, health behaviours, psychosocial work environment and sociodemographic, work and employment characteristics to predict commute mode choices. Prediction models were developed for active and passive commuting mode. We aimed to offer some guidelines for policy-makers aiming to encourage sustainable commuting practices.

## Materials and methods

### Participants

This study was nested within the Finnish Public Sector (FPS) study. The FPS study has been described in detail previously.^21 22^ The study population (n=58 971) included employees of four Finnish cities, with survey data collected from employees during September–October 2020. A total of 42 574 employees (72%) responded. The sample comprised approximately 10% of all public sector employees in Finland in 2020. The majority (84%) of participants were women, which is typical in Finnish public sector. In 2020, the most common occupations in the Finnish municipal sector were those related to healthcare, social services and education, representing nearly 50% of all occupational groups. In the FPS study, filling up the questionnaire is voluntary. The voluntariness is clearly stated in the cover letter of the questionnaire (information to study participants). We also inform the participants that filling out the questionnaire is considered consent to participate. Additionally, we ask for informed consent to link survey data with register data.

Analyses were conducted separately for summer weather and winter weather. Short distance, defined as 5 km or less, was chosen because long commuters cannot be expected to switch similarly from driving to walking or cycling. Thus, employees with a commuting distance of 5 km or less are the core group that could be encouraged to adopt an active commuting mode. Full-time remote workers and part-time workers were excluded because they do not commute to the workplace daily. We performed a multiple imputation with one imputed dataset using the R package Mice (multivariate imputation by chained equations) for the missing predictor data. We used only one imputed dataset because using multiple datasets with LASSO would result in multiple sets of predictors that would differ from each other and could not be readily pooled. The level of missing data before imputation is shown in online supplemental figure 1. The final analytical sample consisted of 10 983 participants.

**Figure 1:**
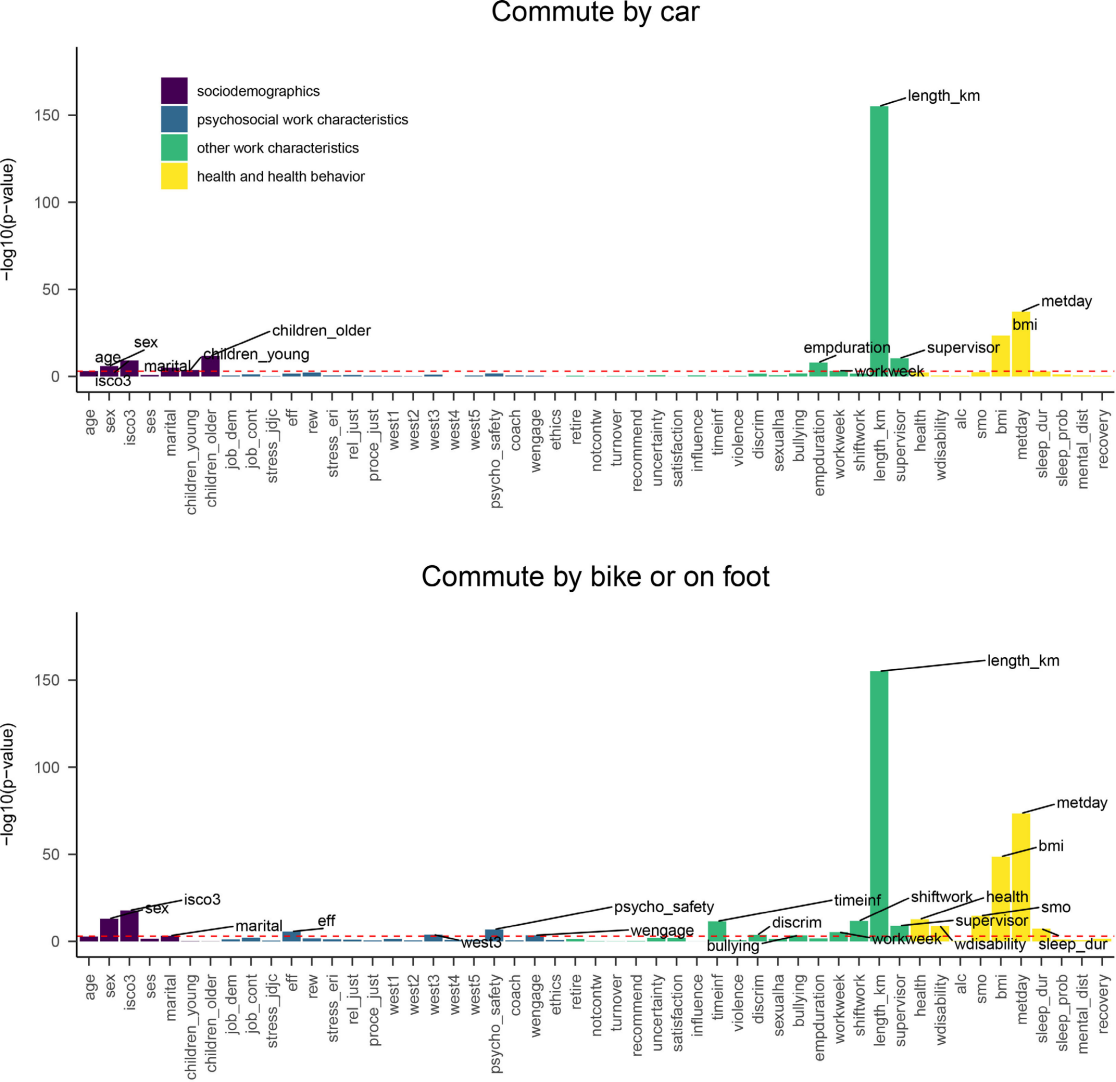
Bivariate associations between predictor items and commute mode choice for passive and active commuting during summer weather. The individual items are grouped with colours to indicate themes. The horizontal red line indicates Bonferroni corrected statistical significance (p=0.05/87=0.0006). Predictors with the highest −log10 (p values) are labelled.

### Measures

The outcome variable, commuting mode, was asked with the following question: “How often do you commute in the summer weather by (1) walking; (2) cycling; (3) public transport with 1000 m or more of walking or cycling; (4) public transport with less than 1000 m of walking or cycling; (5) private car use, either as a driver or a passenger?” The same question was repeated for winter weather. Categories 1–2, walking and cycling, were considered as active commute modes, and five private car use, as passive commute modes. Categories 3 and 4 were omitted from the analysis, as they cannot clearly be classified into active or passive. Response options were as follows: 1=daily or almost daily; 2=a few times a week; 3=once a week; 4=less than once a week; 5 = never.^22 23^ Option 1=daily or almost daily was chosen as the primary outcome variable because it is the most accurate indicator of the main travel mode. For example, choosing option ‘2=a few times a week’ as the outcome for predicting active commuting could mean that the respondent may drive more than 50% of the week to work. However, we ran the additional analysis for active commuting with an outcome of option ‘2=a few times a week’.

The predictor variables were the 87 other questions in the FPS survey on sociodemographic characteristics, health status, lifestyle habits, psychosocial work environment factors and leadership. Most items had a five-point response scale from 1=strongly agree to 5=strongly disagree. A detailed description of the variables is provided in online supplemental appendix 1, but a short description of key predictors follows.

Commute length was asked with an open question: ‘How long is your one-way commute from home?’

The survey included several questions about sociodemographic characteristics: sex, age, type of job contract, occupational title (ISCO-coded=International Standard Classification of Occupations), job tenure, working time (full or part-time; day work or shift work; years in shift work). Sex and occupation were collected from the employers’ registers.

The survey included eight items on the team’s psychological safety, for example, ‘My work is valued at my workplace’, ‘Our workplace is supportive’ and with reverse coded items: for example, ‘Bullying occurs in our workplace’, ‘People on sick leave are easily labeled as truants’. Job demands consisted of five items and job control of nine items.^24 25^ Effort at work was measured with one question: ‘How much do you feel you invest your abilities and resources into your work?’ and rewards with three items.^26^ Seven items measured worktime control.^27 28^ Job insecurities included five items, and changes at work two items. Experiences of organisational justice were measured with seven items of procedural justice, and six items of relational justice.^29^ Supervisor support to employees included four items, and work unit support to supervisor four items. *Worktime control* (seven items) was measured using a questionnaire in which the participants were asked to evaluate how much they could influence the following aspects of their working time: length, starting and ending times, breaks and handling of private matters during the workday, scheduling of work shifts, vacations and paid days off, and the taking of unpaid leave.^27 28^ All these measures of psychosocial work environment were scaled from 1 to 5, with 1 indicating ‘very much’ and 5 indicating ‘very little’.

Participants also reported whether they had had a performance appraisals/career development discussion with their supervisor within the preceding 12 months, and whether they perceived the discussion as useful.

*Physical activity* was measured with questions on average weekly hours of physical activity or exercise during leisure time or commuting within the previous 12 months, with varying intensity corresponding to walking, brisk walking, jogging and running. The response categories were as follows: <30 min, 1 hour, 2–3 hours and >4 hours. We used the following scales for calculations: <30 min = 15 min, 1 hour=45 min, 2–3 hours=2.5 hours and >4 hours = 5 hours. The time spent on activity at each intensity level in hours per week was multiplied by the average energy expenditure of each activity and expressed in MET. Physical activities evaluated to correspond to walking, brisk walking, jogging and running were given MET values 3.5, 5, 8 and 11 MET, respectively.^30^ We treated the variable as a continuous variable so that higher MET hours per day indicated higher physical activity and lower hours less activity.

*Self-reported body mass index* (weight in kg divided by height in m^2^) was dichotomised as less than 25 (non-overweight) and 25 or more (overweight).^31^ Body mass index is a measure commonly used as an indicator of overweight and obesity and is a risk factor for various health conditions.

### Statistical analysis

We built four models: two models for passive commuting in summer and winter weather and two for active commuting in summer and winter weather. The aim was to identify the individual-level predictors of commuting daily by car (passive commuting) and cycling or walking (active commuting). The reason for combining cycling and walking into a single outcome was that the proportion of daily walkers with commute lengths of at least 2 km in the sample was small (5.7%). In Finland, walking reduces to 15% when the journey distance is more than 3 km.^3^ The outcomes for the four models were model (1) daily driving (yes, no); and (2) daily cycling or walking to work (yes, no). These four models were analysed separately for summer and winter weather.

We used bootstrap-enhanced least-absolute-shrinkage-and-selection-operator (lasso) with logistic regression for modelling. The main advantage of the lasso model compared with more conventional regression models is its capability to minimise prediction error and to produce a parsimonious (or sparse) model. Basically, the lasso is standard regression with an l₁-norm penalty. Because of the l₁-penalty, the lasso does variable selection and shrinkage. Lasso forces the sum of the absolute value of regression coefficients to be less than a fixed value dependent on a parameter lambda. When the lambda increases, lasso reduces certain regression coefficients to zero, leaving only the most important predictors for the model.^32^

Before developing the parsimonious prediction models, we split the data with a 75/25 split to training and test datasets stratifying for the outcome, and standardised all predictors for lasso. Then, using bootstrap-enhanced lasso with logistic regression and 10-fold cross-validation, we searched for the optimal lambda value for selecting our predictors.^33^ The predictors selected by regular lasso may vary depending on the sample and the correlation strength of the candidate predictors. With bootstrap-enhanced lasso, the final selected predictors are present in a set proportion of the bootstrap replications. We used 100 bootstrap replications, set the threshold for predictor selection to 95% and used a lambda value that was one SE from the optimal value instead of selecting the optimal lambda. This widely used approach allowed us to get a more parsimonious prediction model while retaining reasonable accuracy compared with the model using the optimal value.^34 35^

We then fit a model using the predictors retained from the bootstrap-enhanced lasso model to the test dataset to get CIs for our estimates. Using that model, we compared the predictions against the observed cases, plotted a Receiver Operating Characteristic curve (ROC), and computed the area under the curve (AUC). AUC is equal to Harrell’s concordance index (C-index)^36^ and has a range from 0.5 (no predictive ability) to 1 (maximum predictive ability). C-index under 0.7 represents poor, 0.7–0.8 good and >0.8 strong discrimination ability.

**Figure 2:**
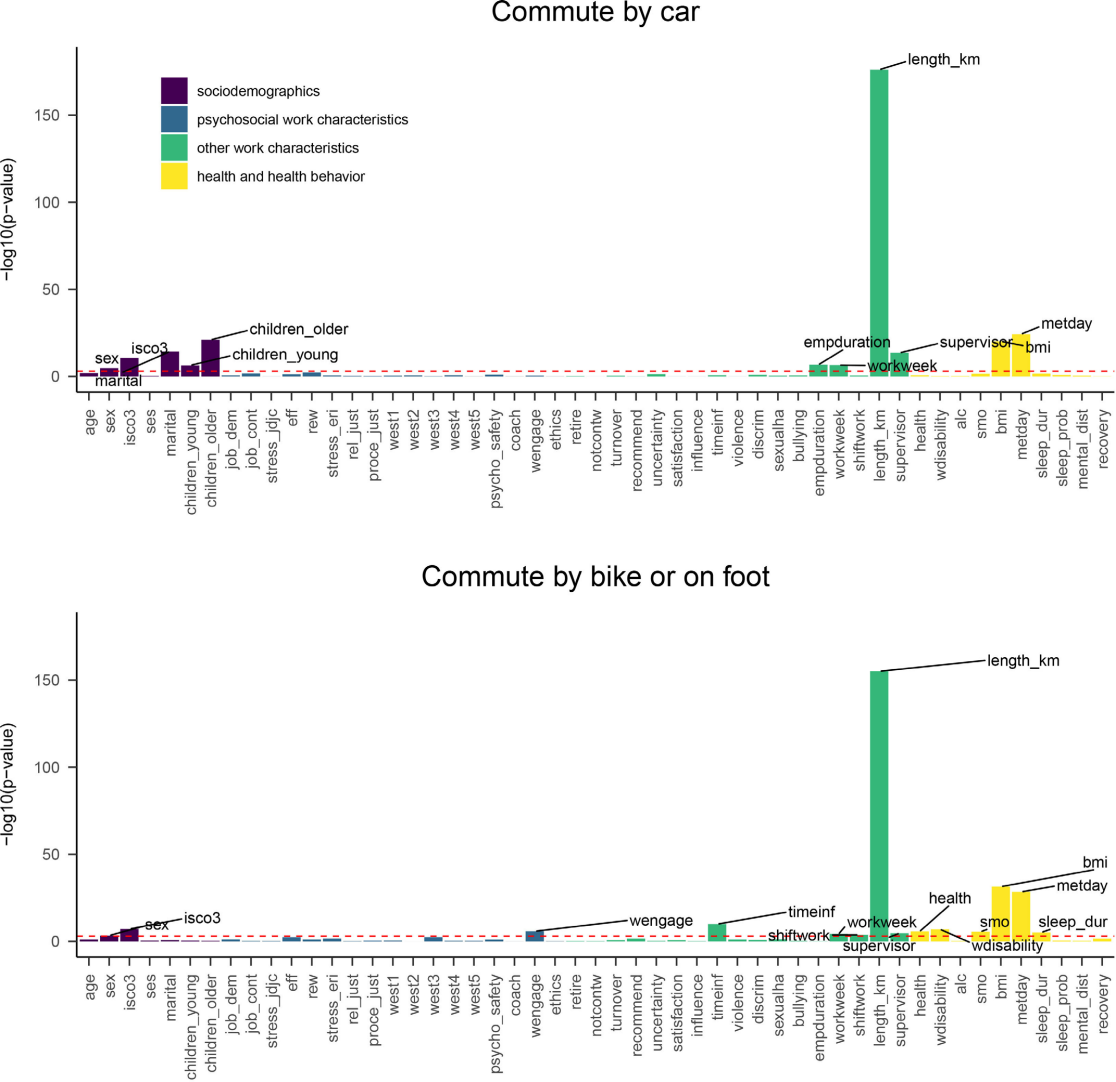
Bivariate associations between predictor items and commute mode choice for active commuting in winter weather. The individual items are grouped with colours to indicate themes. The horizontal red line indicates Bonferroni corrected statistical significance (p=0.05/87=0.0006). Predictors with the highest −log10 (p values) are labelled.

**Figure 3:**
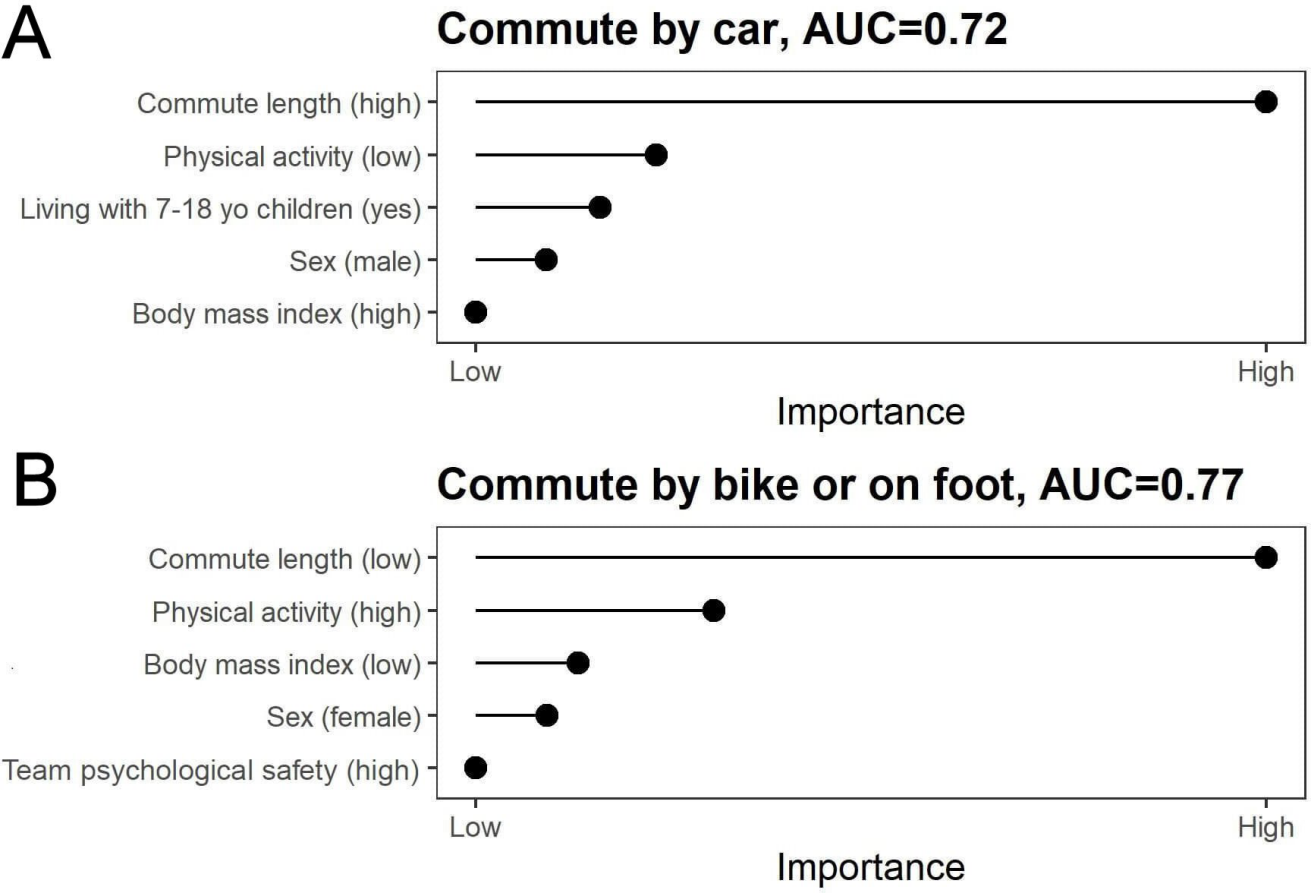
The remaining predictor variables in the main Lasso models. (A) Commuting by car and (B) commuting by bike or on foot in summertime.

**Figure 4:**
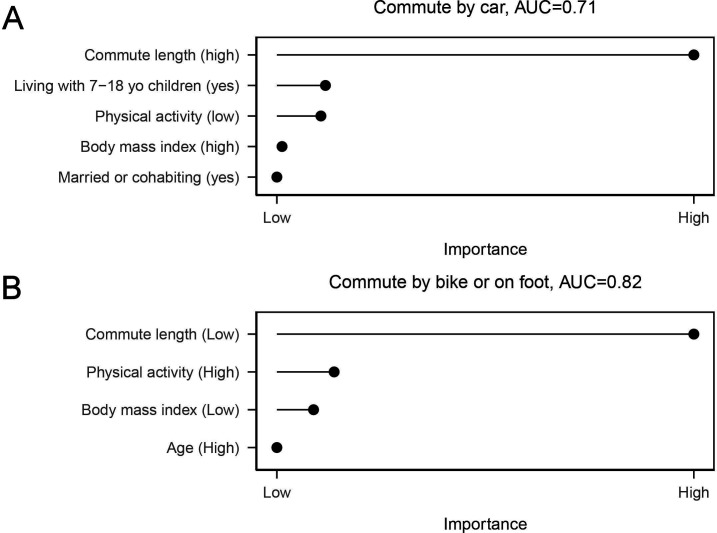
The remaining predictor variables in main Lasso model. (A) Commuting by car and (B) commuting by bike or on foot in the winter weather.

As a sensitivity analysis, we ran an additional six different models. First, four models for active and passive commuting in summer and winter weather without the physical activity (MET) measure. This was done because the measure’s wording did not specify whether the physical activity was related to commuting or leisure time. Second, we ran two models with an outcome of cycling or walking a minimum of a few days a week in summer and winter weather to see whether this affected the results. The tables and figures for all the sensitivity analyses are presented in online supplemental tables 1–4 and figures 2–7.

**Table 1:** Characteristics of the study population (n=10 983)

Characteristic	N (%)
Sex %	
Women	9201 (84)
Men	1782 (16)
SES %	
Upper or lower-level non-manual employee	8051 (73)
Manual worker	2932 (27)
Supervisor position, n (%)	
No	9949 (91)
Yes	1034 (9)
Working time n, (%)	
Full-time	8424 (77)
Part-time	2559 (23)
Commute by car, n (%)	
Less frequently	8802 (80)
Daily	2181 (20)
Commute by walk/bike, n (%)	
Less frequently	4844 (44)
Daily	6139 (56)

**Table 2:** The predictors of summer weather commute mode choice

Predictor	Commute by car		Predictor	Commute by bike or on foot	
	OR	95% CI		OR	95% CI
Commute length (km) (high)	3.86	3.41, 4.36	Commute length (km) (low)	0.13	0.12, 0.15
Physical activity (MET) (high)	0.54	0.47, 0.62	Physical activity (MET) (high)	2.20	1.97, 2.46
Living with 7–19 years old children* (yes)	1.58	1.40, 1.77	Body mass index (kg/m^2^) (low)	0.64	0.58, 0.71
Sex† (male)	1.63	1.41, 1.89	Sex† (female)	0.60	0.53, 0.69
Body mass index (kg/m^2^) (high)	1.34	1.20, 1.50	Team’s psychological safety (high)	1.25	1.13, 1.56

The ORs with their 95% CI for continuous variables represent the effect of each 2 SD increase.

* Reference category=no.

† Reference category 1=female.

**Table 3:** The predictors of winter weather commute mode choice

Predictor	Commute by car		Commute by bike or on foot		
	OR	95% CI	Predictor	OR	95% CI
Commute length (km) (high)	4.03	3.59, 4.53	Commute length (km) (low)	0.06	0.53, 0.59
Living with 7–18 years old children* (yes)	1.55	1.39, 1.73	Physical activity (MET) (high)	1.82	1.63, 2.04
Physical activity (MET) (low)	0.64	0.56, 0.72	Body mass index (kg/m^2^) (low)	0.60	0.54, 0.68
Body mass index (kg/m^2^) (high)	1.35	1.22, 1.51	Age (high)	1.35	1.21, 1.50
Married or cohabiting† (yes)	1.39	1.23, 1.57			

The ORs with their 95% CIs for continuous variables represent the effect of each 2 SD increase.

* Reference category=no.

† Reference category=no.

All analyses were performed using R V.4.1.2 (bolasso (0.1.0) and glmnet (4.1-4)).

### Patient and public involvement

Patients or members of the public were not involved in the research’s design, conduct, reporting or dissemination plans.

## Results

Descriptive characteristics of the study population are shown in table 1. Of the participants, 84% were women, corresponding to the sex distribution in the FPS. The mean age was 46 years (SD 11), and 9% worked in a supervisory position.

Bivariate associations between all items with all four outcomes are presented in figure 1 and figure 2. Most of the statistically significant bivariate associations were related to commute length, health and health behaviour, team’s psychological safety, efforts put to work and sociodemographic characteristics.

Commuting either passively by car or actively by foot or bicycle both during summer and winter weather was best predicted using 8 variables of the 87 original predictors. These predictors are shown in figure 3 and figure 4. The model for winter weather active commute had a strong discriminative ability, AUC=0.82. A good discriminative ability, AUC=0.71, was observed also for commute by car in winter weather. Also, models for summer weather active and passive commute had good discriminative abilities, AUC=0.72 for commute by car, and 0.77 for commute by bike or on foot. The ROC curves for the four main models are presented in online supplemental figures 8 and 9. The individual-level predictors for commute modes, and their ORs with 95% CIs for all models are shown in table 2 and table 3.

The probability to commute by car in summer weather was higher with longer commute length. Other factors associated with a higher probability of commuting by car were low physical activity, living with children from 7 to 18 years old, male sex and higher body mass index. The probability to walk or cycle to work during summer weather was higher with shorter commute length, higher physical activity, lower body mass index, among women and those reporting higher team psychological safety (table 2).

The probability to commute by car in winter weather was higher with longer commute length, living with children from 7 to 18 years old, lower physical activity, higher body mass index and among participants who were married or cohabiting. The probability to walk or cycle to work during winter weather was higher with shorter commute length, higher physical activity, lower body mass index and higher age (table 3).

A sensitivity analysis for summer weather commuting by car without predictor variable of physical activity resulted in a model with three predictors: longer commute length, higher body mass index and living with children aged 7–18 years. The predictive performance of the more parsimonious model was similar (AUC 0.72). The associations and ROC curves of the sensitivity analyses are presented in the online supplemental table 1 and figure 2. Sensitivity analysis for summer weather commuting by bike or on foot without physical activity variable resulted in a model with six predictors: shorter commute length, lower body mass index, not smoking, being a woman, high effort at work and higher team psychological safety. The model performance was worse (AUC 0.76). The associations and ROC curves are presented in the online supplemental table 1 and figure 3.

Sensitivity analysis with an outcome of a minimum of a few days walking or cycling per week in summer weather produced a model with a similar AUC of 0.77. The model included five predictors: shorter commute length, high physical activity, low body mass index, not smoking and marriage or cohabitation (online supplemental table 2 and figure 4). The final sensitivity analysis for winter weather active commuting a minimum of a few days per week produced a model with five predictors: shorter commute length, higher physical activity, lower body mass index, being a non-smoker and having better worktime control. The model performed weaker than the main model (AUC 0.81) (online supplemental table 4 and figure 7).

## Discussion

In this study, we developed prediction models for commuting modes on short journeys using a sizeable public sector workplace survey of 10 983 employees. We built four models: two for active commuting modes of walking and cycling during summer and winter weather and another two for passive commuting by car during summer and winter weather. The models were good at identifying characteristics of individuals who are likely to choose either an active or a passive mode for commuting short distances.

All models resulted in four or five predictors. Passive commuting by car in summer weather was associated with higher journey length, lower physical activity, living with 7–18 years old children, male sex and higher body mass index. Active commuting in summertime was associated with shorter commute length, higher physical activity, lower body mass index, female sex and better team psychological safety. Passive winter weather commuting was associated with longer commute length, living with 7–18 years old children, lower physical activity, higher body mass index and marriage or cohabitation. Active commuting in wintertime was associated with shorter commute length, higher physical activity, lower body mass index and higher age. The results were largely replicated in multiple sensitivity analyses.

### Previous research on the identified predictors of commute mode

Although the commute length was limited to short commutes of 5 km or less, the strongest predictor of commuting mode choice was length in all four models. This finding is supported by previous studies.^7^,^10^ Shorter commute was associated with walking or cycling, and longer commute with driving a car. While more than half of the study population (56%) cycled or walked every day to work in summer weather, still 20% of the participants drove to work daily despite the short journey, which takes a maximum of 30 min by cycle.

Physical activity and body mass index were linked to both active and passive modes of commuting in all models. Previous studies also demonstrate a robust association between these factors and commute mode.^37^ The negative association between body mass index and active commuting is consistent with previous cross-sectional studies.^38^,^40^ A longitudinal study^41^ showed that switching from passive commuting to active commuting and maintaining active commuting can help control body weight among working-aged adults of both sexes. As the prevalence of overweight and obesity increases,^40 42 43^ the role of active commuting may prove an effective weight control strategy in working-age adults. E-bikes could be a promising alternative, as they lower the barrier to start cycling and help people maintain cycling, contributing to meeting physical activity guidelines. Although e-bikes require less effort than regular bicycles, several studies have found that they still provide moderate-intensity physical activity.^44^

One consistent finding was that family situation was associated with passive commuting. Living with children aged from 7 to 18 was associated with a higher likelihood to commute by car during both summer and winter weather. Living with a partner was associated with a higher likelihood of driving during winter weather. Earlier findings regarding family situations and commuting modes have been varying. Although there are also opposite results from high cycling countries, Germany and Netherlands,^5 45 46^ several studies indicate that parenthood, of school-aged children in particular, is associated with increased car use and decreased active travel.^11 17 40 47^ This effect is more pronounced in men than women.^48^ Possible explanations exist for the association between living with school-aged children and driving. Time constraints and greater necessity for trip chaining, such as dropping off or picking up children from schools or daycare centres, are direct effects of parenthood affecting commute mode choice.^48 49^ There are also indirect effects, such as increased car ownership, that may significantly impact commute mode choice.^20 47^ Living in a common law or marital relationship can also increase the likelihood of purchasing a car, as the financial situation is often significantly better. Owning a car, in turn, can partly explain the connection to choosing passive commute mode.^6 19 20^

Although sex did not emerge in the winter weather prediction models, it explained cycling and driving in the summer weather, with men being more likely to drive and women to cycle. The result agrees with previous research showing that women are less dependent on private cars than men are.^6 9 11 12^ In Finland, men drive more and longer journeys than women in all age groups.^6^ One explanation may be that men, more often than women, have the identity of a driver. Thus, men use the car in the household. Previous research showed that people with car-related self-image who value travel time, comfort and freedom,^50^ or status power,^51 52^ are more likely to commute by car.^16^

Our study finds that older employees are more likely to cycle to work during winter. Countries with higher cycling rates tend to have more older cyclists than low-cycling countries.^5 53^ Our research cannot address the factors that explain the observed relationships between the variables. However, as parents of young children are living through the busiest years and often need to run errands during their commute, they may, therefore, rely on cars to save time. In contrast, older working-age individuals who have passed the busiest years have less urgency and more time for commuting by bicycle.

We found no previous research on the role of psychosocial or other work characteristics in commute mode choices. Our data included several such measures. One of these measures, namely, team psychological safety, remained in the final prediction model. Higher team psychological safety was associated with active commuting in summer weather. When the work team is perceived as supportive and helpful, people may have lower anxiety and stress levels,^54^ indicating a positive mental state, and thus they may be more willing and have more energy to cycle or walk to work. A psychologically safe work environment may foster a healthy work–life balance, motivating employees to take care of their overall well-being, including physical activity. Moreover, a strong sense of community and teamwork often prevails in workplaces with high psychological safety.^54^ Employees may feel supported and encouraged by their peers to engage in active commuting, whether through group initiatives or informal encouragement. A previous study suggests that for women, social environment may play a significant role in choosing the commute mode, whereas men perceive bike-friendly facilities (eg, changing rooms, showers, bike storage) as more important determinants.^55^ Active commute might also improve perceptions of team psychological safety. In an intervention study, the active travel group indicated more positive organisational behaviour and positive feelings than the passive travel group.^56^

### Strengths and limitations

The strengths of this study include a large sample from a cohort representative of FPS employees across various occupations. The FPS is the most extensive survey examining the work and health of public sector staff in Finland. The response rate was high, 72%. Our analytical sample (table 1) represented the survey population well, in which the proportion of women was 78.9 %, and the mean age was 46 years, with a SD of 11. Moreover, considering both summer and winter weather, commute distance and frequency of the outcome measure, we gained more detailed insights into commute mode choices and enhanced the robustness of our main findings. Additionally, to our knowledge, the study included factors associated with commute mode, such as the psychosocial work environment, which have not been previously explored.

However, the study also has limitations. All survey items were self-reported and therefore subject to recall and/or social desirability bias. Furthermore, while we did have more than 1700 men in our sample, most participants were women, which is typical in the FPS. Thus, the generalisability of our findings to male-dominated private sector employees, other environments, societies and cultures remains uncertain. Despite including many predictors, we lacked information on employer-provided parking facilities, commute infrastructure and the environment, which could influence commuting choices. Previous evidence indicates that eliminating free workplace parking effectively reduces car-based commutes, as incentives, convenience and habit encourage car use.^57^,^59^ In the future, it might also be useful to separate walkers and cyclists in the analyses, as the correlates may differ. Lastly, the data collection occurred in September–October 2020, after the outbreak of the COVID-19 pandemic. The pandemic may have led to more people commuting by car or active modes than normally. Nevertheless, our results mainly align with previous findings of predictors of commute mode choice from both pre-pandemic^7 37 41^ and post-pandemic^23^ pandemic periods.

### Conclusions

We created prediction models to identify the characteristics of individuals who are likely to choose a bike and those of a car commuter. Our prediction models, with eight predictors, were accurate at identifying the characteristics of these individuals who were active or passive commuters in summer and winter weather.

Alongside the better-known correlates, such as commute length and physical activity, we discovered a new, previously unidentified psychosocial factor associated with active commuting: psychological safety. Better team psychological safety was linked to daily active commuting during the summer weather. Notably, this factor can be modified with interventions. Workplaces with high psychological safety are likely also committed to the overall well-being of their employees, including healthy work–life balance and promotion of physical activity. Psychological safety may be accompanied by empowerment and autonomy.^54^ Employees in control of their work and personal lives may be more inclined to take proactive measures to enhance their health and well-being, including opting for active commuting.

Our models can help inform intervention studies. If our findings can be replicated in other cohorts and are supported by intervention studies, policy-makers and urban planners can justify investments in biking routes and facilities, and employers in workplace environments that support active commuting, ultimately fostering healthier and more sustainable communities.

## supplementary material

10.1136/bmjopen-2023-080276 online supplemental file 1

10.1136/bmjopen-2023-080276 online supplemental file 2

## Data Availability

No data are available.
