# Supplementary material for: Development of a multifactorial prediction model for commute mode choice in 10 983 Finnish public sector employees: a cross-sectional study
Source: BMJ Open. 2024 Oct 16;14(10):e080276. doi: 10.1136/bmjopen-2023-080276 (PMC11487787; doi:10.1136/bmjopen-2023-080276)
Supplement: online supplemental file 1 [file bmjopen-14-10-s001.pdf]

Web appendix 1.

Development of a Multifactorial Prediction Model for Commute Mode Choice in 10,983 Finnish Public Sector Employees: A Cross-sectional Study

Detailed description of variables included in the prediction models

Sociodemographic variables

We had information on the respondents' *Age* (as a continuous variable), *sex* (man, woman), and occupation collected from the employers' registers. We also had information on the type of job contract (temporary/permanent) and occupational title (ISCO-coded), Job tenure (years with employer and years in the current position), and working time (full or part-time; day or shift work; years in shift work).

Marital status (having a partner; yes/no), *children aged 0-6 years* (yes/no), *children aged 7-18 years* (yes/no), and *individual commute length* (as a continuous variable; one-way commute distance in kilometers) were self-reported.

Work unit level variables were created as: average age; percentage of women; percentage of temporary employees; percentage of managers, senior officials, and professionals (ISCO codes 1-2); percentage of manual workers (ISCO codes 5-9); turnover percentage (share of new employees as compared to previous survey); size of the work unit; average job tenure; average years in current job; percentage of part-time workers, the percentage in day job; average years in shift work.

Work characteristics

The survey included measures of *job demands and job control*[1,2]. Job demands were measured with 5 items: time pressures and deadlines, lack of time to do what was expected, and work overload. The job control scale combines two concepts: skill discretion (the opportunities of an individual to develop his or her special abilities within the job, 6 items) and decision authority (individual's abilities to be part of the decision-making process within the organization, 3 items).

For the *Team's psychological safety,* the survey included eight items "My work is valued at my workplace", "Our workplace is supportive", "people at our workplace can really be trusted", "there is an open camaraderie at our workplace characterized by mutual helpfulness", and with reverse coded items: "Bullying occurs in our workplace", "gossip and jealousy occur at our workplace", "I face discrimination at our workplace", " people on sick leave are easily labeled as truants".[3]

Efforts and rewards at work (4 items): Effort was measured with one question ("How much do you feel you invest in your job in terms of skill and energy?") and reward was assessed with three questions about feelings of getting a return from work in terms of (1) income and job benefits, (2) recognition and prestige, and (3) personal satisfaction. The response format was a five-point scale from 1 =very much to 5=very little.[4] The ratio of the self-assessed effort score to the mean of the self-assessed reward scores formed the *effort-reward imbalance* (ERI). Scores above the median was defined as high ERI.

Worktime control (7 items) was measured using a questionnaire in which the participants were asked to evaluate on a scale from 1 (very much) to 5 (very little) how much they could influence the following aspects of their working time: length, starting and ending

times, breaks, and handling of private matters during the workday, scheduling of work shifts, vacations and paid days off, and the taking of unpaid leave.[5,6]

Job insecurities were measured with 5 items: 'Does your job involve the following insecurities': the threat that some work tasks will be terminated; involuntary transfer into another work tasks; the threat of temporary lay-offs; the threat of permanent lay-offs; the threat of excessive workloads. The scale was from 1=very much to 5=very little.

Changes at work were measured with 2 items: 'When you think about all the changes that have happened in your work during the last 12 months, how would you describe those from your own point of view?' with a scale from 1=mostly positive to 7=mostly negative. We also enquired whether the respondent felt she/he could take part when changes were planned with a response scale 1=I have very much influence over the changes; 2=I have some influence over the changes; 3=Most often the changes occur unexpectedly, I don't have influence over them.

Leadership and management

Procedural justice (7 items): The scale considers whether the decision-making procedures at the workplace are accurate, correctable, consistently applied, and whether the procedures include opinions from the people involved.[7]

Relational justice (6 items): The scale includes items evaluating whether the supervisors use kindness and consideration, are truthful, and can suppress personal biases.[7]

Supervisor support (4 items): The scale includes items evaluating the extent to which the supervisor supports and encourages, rewards from good performance, trusts, and encourages employees to educate and develop themselves in their work.

In *support from the work unit to supervisor* (4 items), participants were asked to evaluate the extent to which employees perceive employees to have a role in successful leadership, inform their supervisor on work-related matters, value the competence of their supervisor, and support their supervisor.

Performance appraisals/career development discussions were measured with two items: having had such a discussion within the last 12 months (1=No, 2=Yes), and whether the discussion was perceived useful (1=Useful, 2=Not useful, but not totally useless, 3=Useless).

Work unit/team climate

Team climate (14 items): The work unit cooperation and interaction was measured using the short version[8] of the Team Climate Inventory (TCI)[9]. TCI conceptualizes team climate into four dimensions: participations safety (4 items), support for innovation (3 items), vision (4 items), and task orientation (3 items).

Discrimination at work was measured with a single item: Is there discrimination due to age, gender, education, opinion, status, origins, language, religion, believes/convictions, political activity, trade union activity, health, disability, sexual orientation, or gender identity/gender expression? (1=No, 2=Yes).

Satisfaction with job, satisfaction with employer, and intentions to leave

Job satisfaction was measured with 5 items: Employees were asked to evaluate the extent to which they were satisfied with their personal growth and development; the feeling of accomplishing something significant; possibilities to think and act independently at work; challenges provided by the job. The scale was from 1=very unsatisfied to 5=very satisfied. In

addition, we asked whether the respondent would recommend the current employer to a friend, with a scale dichotomized into 1=Yes; 2=No.

Retirement intentions were measured with a single item: “Do you see yourself working until your personal retirement age?” with response scale 1=Yes, I see myself working until my retirement age; 2=Yes, I see myself working even after my retirement age; 3=No, I don’t see myself working until retirement age.

Turnover intentions were measured with a single item: “Have you considered changing employer?” with response scale 1=No, I want to keep working for my current employer; 2=Yes, I have considered changing employer; 3=I have already recruited to another employer.

Health and lifestyle

Psychological distress (symptoms of depression and anxiety) was measured with the 12-item General Health Questionnaire, where respondents rate the extent to which they are affected by 12 symptoms of distress. Four or more symptoms were coded as cases.[10]

Sleep problems were measured with the Finnish version of the Jenkins Sleep Scale, which comprises four items: 1) the difficulty to fall asleep, 2) wake up at night, 3) difficulty to stay asleep, and 4) non-restorative sleep. The response format was a five-point scale, ranging from 0 (never) to 5 (almost every night), with higher scores indicating more sleep problems.[11]

Perceived health was a single-item measure “How do you rate your health?” with response options from 1=poor to 5=good. The question is widely used in surveys and recommended for standard indicator of health.[12]

Self-rated work ability was a single-item measure from the Work Ability Index[13,14]: “Let’s assume that your work ability at its all-time best would be given 10 points, and 0 points would indicate that you are completely unable to work. How would you score your current work ability?” Response options ranged from 0 to 10. This single item and the entire Work Ability Index are very strongly associated and showed similar associations with sickness absence health, and symptoms.[15]

Self-reported Body mass index (weight in kg divided by height in m^2) was dichotomized as less than 25 (non-overweight) and 25 or more (overweight).[16] Body mass index is a measure of body fat, commonly used as an indicator of overall health and risk for various health conditions.

Alcohol use was defined through questions on weekly consumption. One drink was approximately equivalent to one unit or one glass of alcoholic drink or 12 g of alcohol. Alcohol use was dichotomized into no use or moderate use (max of 140 g or 11 units for women and 280 g or 23 units for men) versus alcohol use greater than this.[17]

Smoking was dichotomized into current smoker and non-smoker (including never and ex-smokers).[18]

Total Physical activity was measured with questions on average weekly hours of physical activity or exercise during leisure time or commuting within the previous 12 months, with varying intensity corresponding to walking, brisk walking, jogging, and running. The response categories were: < 30 minutes, 1 hour, 2–3 hours, and > 4 hours. We used the following scales for calculations: <30 minutes = 15 minutes, 1 hour = 45 minutes, 2–3 hours = 2.5 hours, and >4 hours = 5 hours. The time spent on activity at each intensity level in hours per week was multiplied by the average energy expenditure of each activity and expressed in MET. Physical

activities evaluated to correspond to walking, brisk walking, jogging, and running, were given MET values 3.5, 5, 8, and 11 MET, respectively.[19]

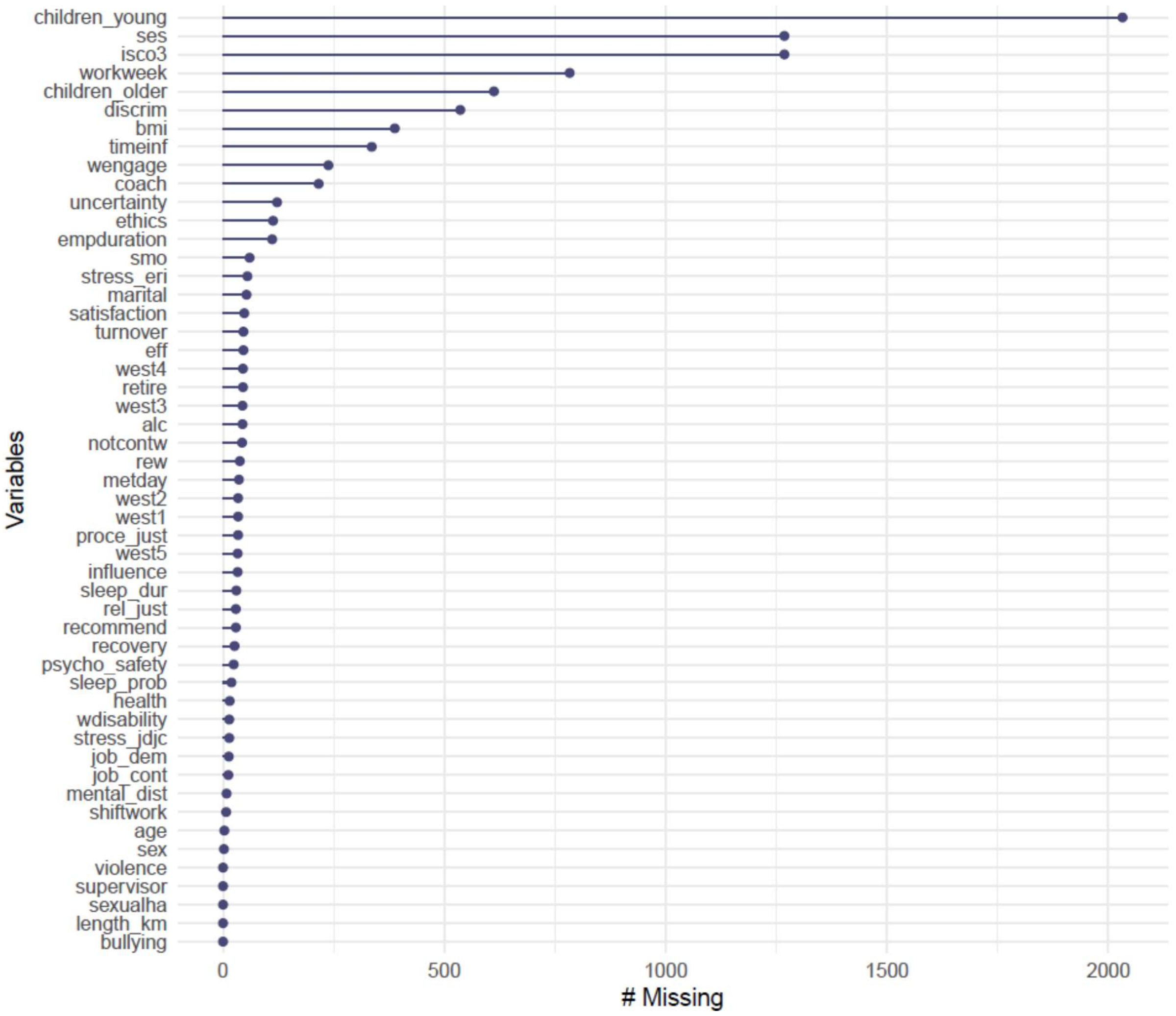

Web figure 1 The level of missing data before imputation

Web Table 1 Predictors of commuting by car and by bike or on foot in summer weather, excluding physical activity. Lasso regression. The odds ratios with their 95% confidence intervals (CI) for continuous variables represent the effect of each two standard deviation increase.

COMMUTE BY CAR			COMMUTE BY BIKE OR ON FOOT		
Predictor	OR	95% CI	Predictor	OR	95% CI
Commute length (high) km	3.79	3.36,4.28	Commute length (low)	0.14	0.13,0.16
Body mass index (kg/m^2) (high)	1.54	1.38,1.71	Body mass index (kg/m^2) (low)	0.55	0.49,0.60
Living with 7-18-year-old children[1] (yes)	1.52	1.36,1.71	Smoking[2] (no)	0.65	0.56,0.76
			Sex[3] (female)	0.73	0.64,0.84
			Efforts (high)	1.20	1.09,1.33
			Team psychological safety (high)	1.20	1.09,1.32

[1]Reference category=no, [2]reference category=no, [3]Reference category=female

Web Table 2 Predictors of commuting by bike or on foot for a minimum of a few days per week in summer weather. Lasso regression. The odds ratios for continuous variables with their 95% confidence intervals (CI) represent the effect of each two standard deviation increase.

	COMMUTE BY BIKE OR ON FOOT	
Predictor	OR	95% CI
Commute length (km) (low)	0.12	0.11,0.13
Physical activity (MET) (high)	2.29	2.02,2.60
Body mass index (kg/m^2) (low)	0.66	0.60,0.74
Smoking[1] (no)	0.67	0.57,0.78
Married or cohabiting[2] (yes)	1.25	1.12,1.39

[1]Reference category=no, [2]reference category=yes

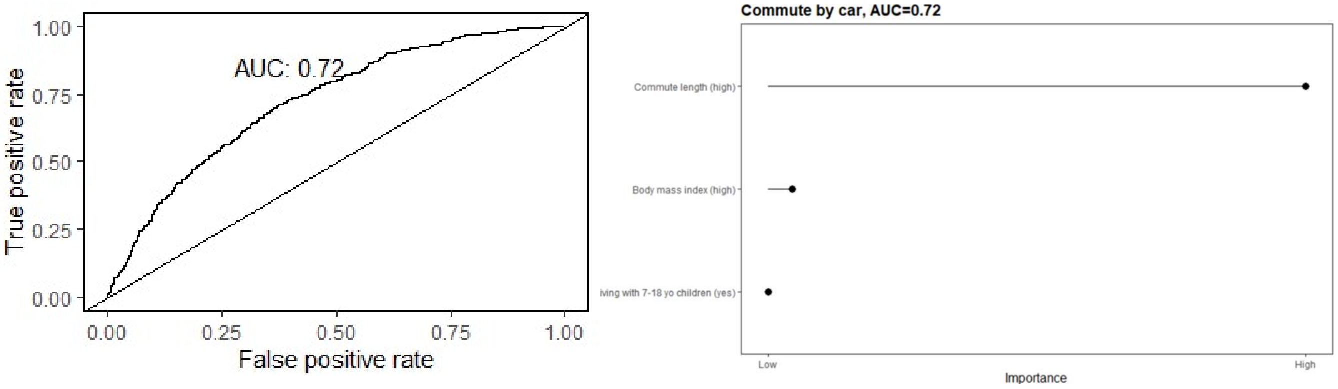

Web figure 2. Model performance of commuting by car without physical activity in summer weather. Panel A: ROC-curve for prediction of commuting by car (95% threshold for predictor selection). Panel B: remaining predictors in the model.

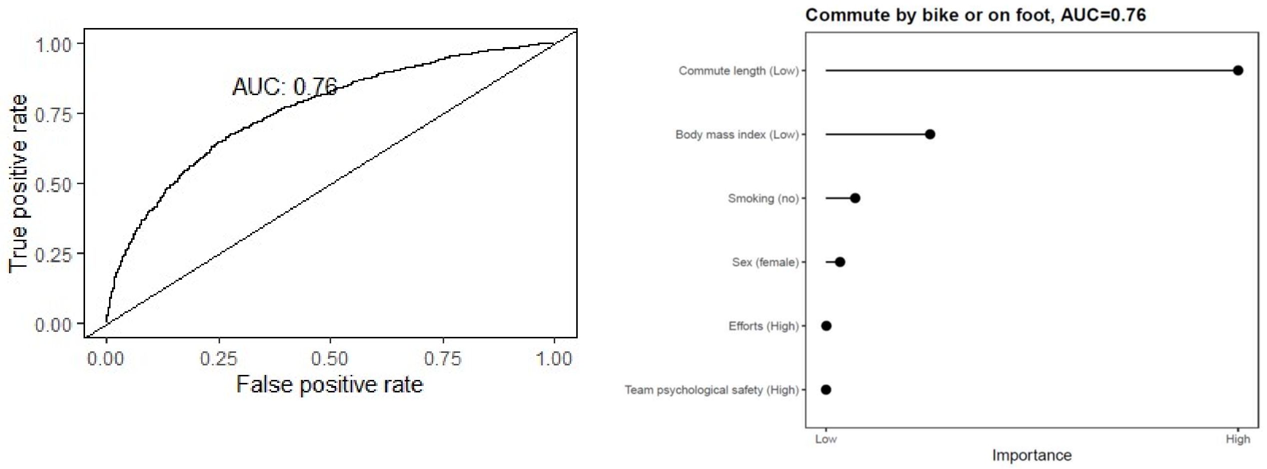

Web figure 3. Model performance without physical activity in summer weather. Panel A: ROC-curve for prediction of commuting by walk or cycle (95% threshold for predictor selection). Panel B: remaining predictors in the model.

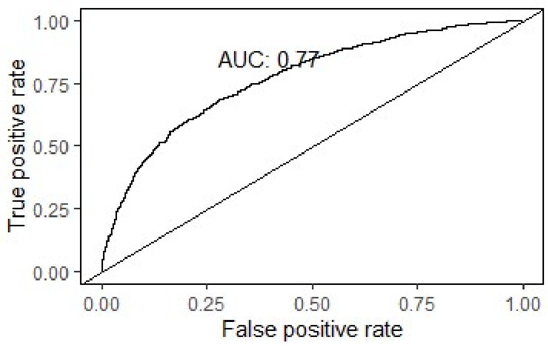

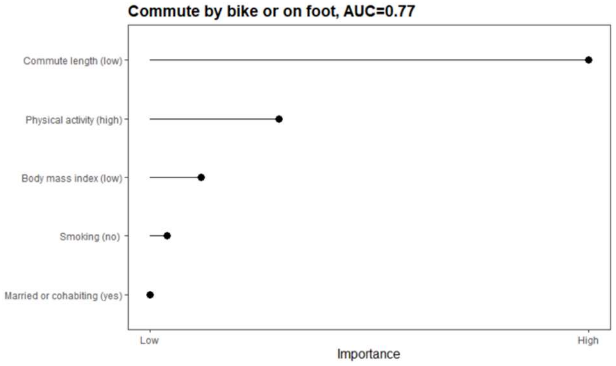

Web figure 4. Model performance for commuting by bike or on foot for a minimum of a few days per week in summer weather. Panel A: ROC-curve for prediction of commuting by bike or on foot (95% threshold for predictor selection). Panel B: remaining predictors in the model.

Web Table 3. Predictors of commuting by car and by bike or on foot in winter weather, excluding physical activity. Lasso regression. The odds ratios with their 95% confidence intervals (CI) for continuous variables represent the effect of each two standard deviation increase.

COMMUTE BY CAR			COMMUTE BY BIKE OR ON FOOT		
Predictor	OR	95% CI	Predictor	OR	95% CI
Commute length (km) (high)	´3.95	´3.52, 4.43	Commute length (km) (low)	0.06	0.05, 0.07
Body mass index (kg/m2) (high)	´1.49	´1.34, 1.65	Body mass index (kg/m2) (low)	´0.54	0.49, 0.61
Living with 7−18 yo children[1] (yes)	´1.51	´1.35, 1.68			
Married or cohabiting[2] (yes)	´1.38	´1.23, 1.56			

[1]Reference category=no, [2]Reference category=no

Web table 4. Predictors of active commuting by bike or on foot in winter weather for a minimum of a few days per week. Lasso regression. The odds ratios with their 95% confidence intervals (CI) for continuous variables represent the effect of each standard deviation increase.

COMMUTE BY BIKE OR ON FOOT

Predictor	OR	95% CI
Lenght (km) (low)	0.07	0.06, 0.08
Physical activity (MET) (high)	´1.96	1.76, 2.20
Body mass index(kg/m2) (low)	´0.63	0.57, 0.71
Smoking[1] (no)	´0.61	0.52, 0.72
Worktime control (high)	´1.27	1.17, 1.41

[1]Reference category=no

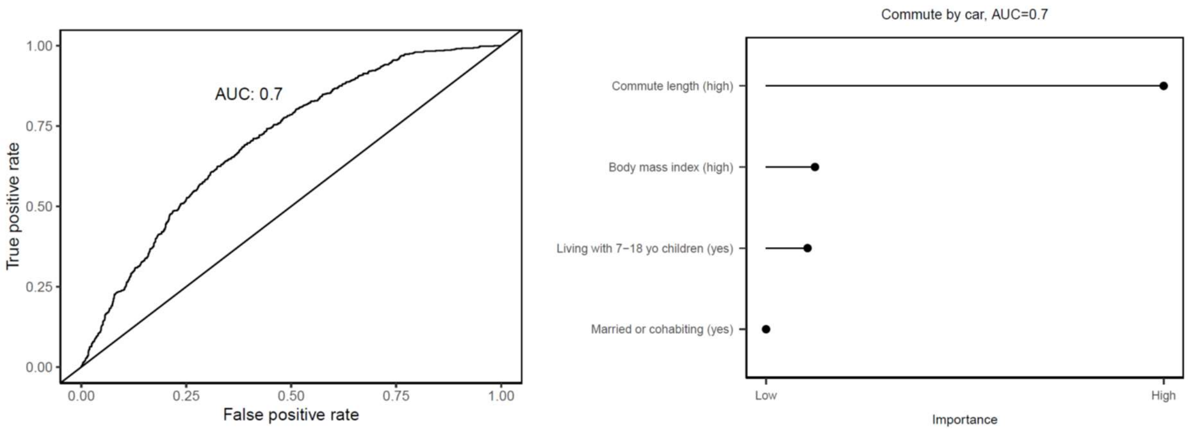

Web figure 5. Model performance of commuting by car without physical activity in winter weather. Panel A: ROC-curve for prediction of commuting by car (95% threshold for predictor selection). Panel B: remaining predictors in the model.

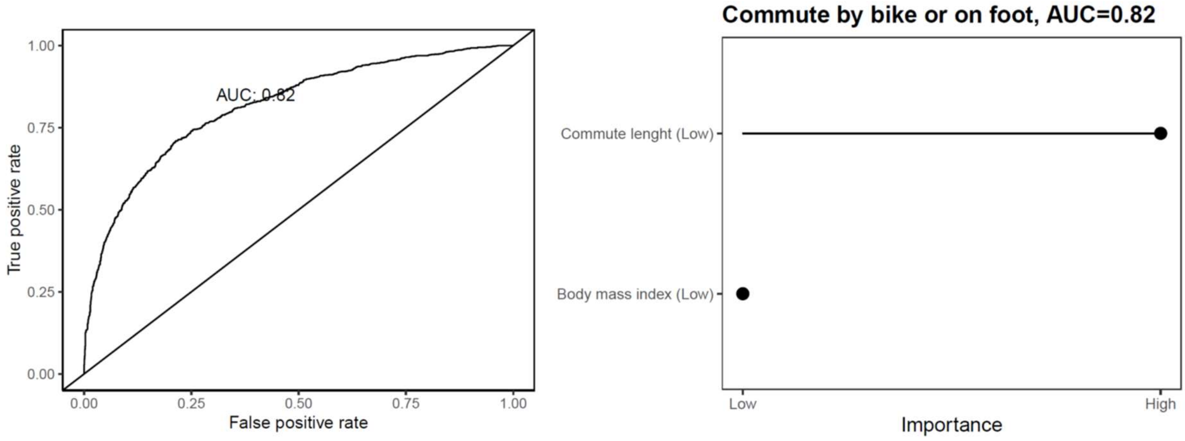

Web figure 6. Model performance for commuting by bike or on foot in the winter weather without physical activity. Panel A: ROC-curve for predicting commuting by bike or on foot in winter weather (95% threshold for predictor selection). Panel B: remaining predictors in the model.

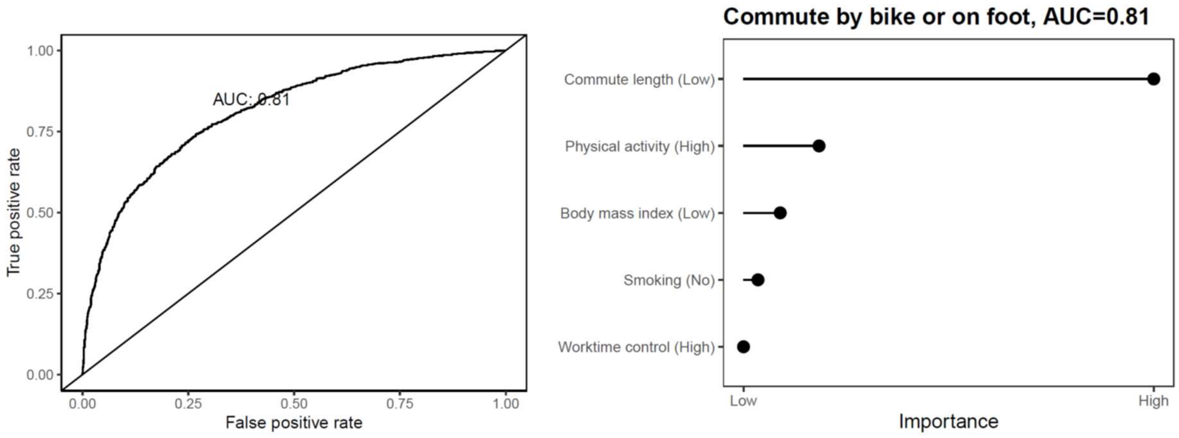

Web figure 7. Model performance for commuting by bike or on foot in the winter weather for a minimum of a few days per week. Panel A: ROC-curve for predicting commuting by bike or on foot (95% threshold for predictor selection). Panel B: remaining predictors in the model.

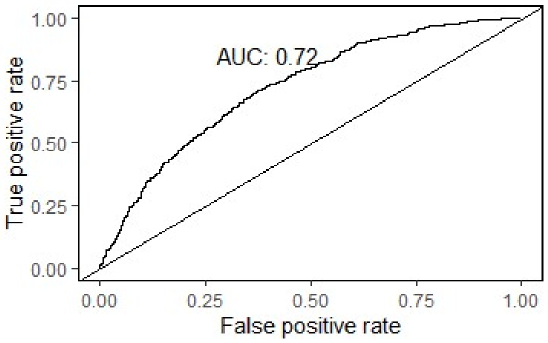

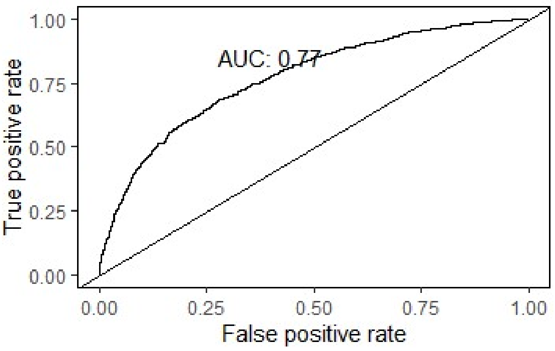

Web figure 8. The model performance for the main models in summer weather. Panel A ROC-curve for the model predicting commuting by car and panel B ROC-curve for commuting by bike or on foot.

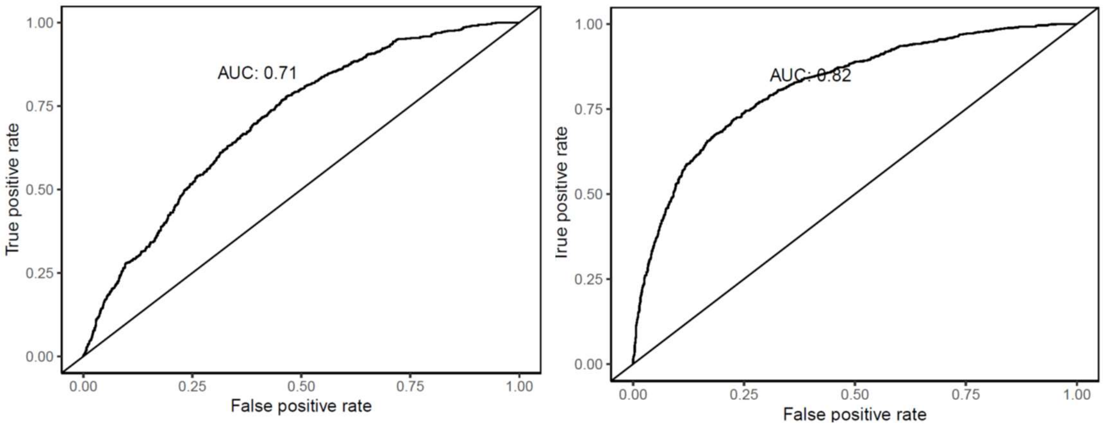

Web figure 9. The model performance for the main models in winter weather. Panel A ROC-curve for the model predicting commuting by car and panel B ROC-curve for commuting by bike or on foot.

Example analytic code used in the study

R-code for precting active commuting:

Code for walking and cycling daily during summer time (walkbikekes=walking or cycling during summer)

```
#source(here("scripts", "0 wrangling.R"))

dyhtkeskp <- dyhtimp %>%

select(-carkes,-cartal, -nocarkes,-walkbiketal,

        -walkbikekes,) %>% # optional outcomes

 #journey distance:

 #filter(length_km<=5) %>%

#standardization of the continuous variables (2 sd)

mutate_if(is.numeric, scale_this)

set.seed(2022)

splits <- initial_split(dyhtkeskp, strata = walkbikekes_c)

train <- training(splits)

test <- testing(splits)

y <- train %>% select(walkbikekes) %>% data.matrix()

x <- train %>% select(-walkbikekes) %>% data.matrix()
```

```
bofit <- bolasso(x=x,y=y, BM=100, kfold=10, family="binomial", implement="glmnet",
        standardize=F)
vars <- selected_vars(bofit, threshold = .95,  select = "lambda.1se") #lambda.1se
model <- as.formula(paste0("walkbikekes_c~",paste(vars$variable[-1], collapse="+")))
newfit <- glm(model,data=train, family="binomial")
wb_or<-tidy(newfit, exp=T, conf.int = T)
vipvalues<-newfit %>% vi(method = "model")
vipvalues
walk5plot<-   ggplot(vipvalues,aes(y=reorder(Variable,   Importance),x=Importance))+   #
color=Sign))  +
geom_point(size=3) +
geom_segment(aes(x=min(Importance), xend=Importance, y=reorder(Variable, Importance),
yend=reorder(Variable, Importance))) +
theme_apa() +
theme(legend.position="none") +
#scale_color_manual(values=c("POS"="red", "NEG"="blue")) +
scale_x_continuous(breaks = range(vipvalues$Importance),
          labels=c("Low", "High")) +
```

```
labs(y="")

test$pred <- predict(newfit, newdata=test)

pred <- prediction(test$pred,test$walkbikekes_c)

perf <- performance(pred,"tpr","fpr")

auc <- performance(pred, measure = "auc")

auc <- auc@y.values[[1]]

auc <- round(auc,2)

auc_aggre_data <- data.frame(x=perf@x.values[[1]], y=perf@y.values[[1]])

#AUC curve!

auc_aggre_data <- data.frame(x=perf@x.values[[1]], y=perf@y.values[[1]])

ggplot(auc_aggre_data, aes(x,y))+

geom_line()+

geom_abline(intercept = 0, slope = 1)+

labs(x="False positive rate", y="True positive rate")+

theme(panel.grid.major = element_blank(), panel.grid.minor = element_blank(),

panel.background = element_blank(), axis.line = element_line(colour = "black"),

panel.border = element_rect(colour = "black", fill=NA))+
```

```
annotate("text",.4,.85,label=paste0("AUC: ",auc))

walk5plot <-walk5plot + ggtitle(paste0("Commute by bike or on foot, AUC=",auc)) +

 #scale_y_discrete(labels=c("Body mass index (low)","Physical activity (high)","Commute length (low)"))

 #scale_y_discrete(labels=c("Sex (female)","Smoking (no)","Body mass index (low)", "Commute length (low)"))

 #scale_y_discrete(labels=c("Work climate (good)", "Sex (female)", "Body mass index (low)","Physical activity (high)","Commute length (low)"))

#scale_y_discrete(labels=c("Work climate (good)","Efforts (high)", "Sex (female)","Smoking (no)","Body mass index (low)","Commute length (low)"))

walk5plot
```

References

1. Karasek, R. A. Job Demands, Job Decision Latitude, and Mental Strain: Implications for Job Redesign. *Adm. Sci. Q.* **24**, 285 (1979).

2. Karasek, R. A. & Theorell, T. *Healthy Work: Stress, Productivity and the Reconstruction of Working Life.*

3. Ervasti, J. *et al.* Prediction of bullying at work: A data-driven analysis of the Finnish public sector cohort study. *Soc. Sci. Med.* **317**, 115590 (2023).

4. Kivimaki, M., Vahtera, J., Elovainio, M., Virtanen, M. & Siegrist, J. Effort-reward imbalance, procedural injustice and relational injustice as psychosocial predictors of health: complementary or redundant models? *Occup. Environ. Med.* **64**, 659–665 (2007).

5. Ala-Mursula, L. Effect of employee worktime control on health: a prospective cohort study. *Occup. Environ. Med.* **61**, 254–261 (2004).

6. Vahtera, J. *et al.* Employee control over working times and risk of cause-specific disability pension: the Finnish Public Sector Study. *Occup. Environ. Med.* **67**, 479–485 (2010).

7. Moorman, R. H. Relationship between organizational justice and organizational citizenship behaviors: Do fairness perceptions influence employee citizenship? *J. Appl. Psychol.* **76**, 845–855 (1991).

8. Kivimaki, M. & Elovainio, M. A short version of the Team Climate Inventory: Development and psychometric properties. *J. Occup. Organ. Psychol.* **72**, 241–246 (1999).

9. Anderson, N. & West, M. A. The team climate inventory: Development of the tci and its applications in teambuilding for innovativeness. *Eur. J. Work Organ. Psychol.* **5**, 53–66 (1996).

10. GOLDBERG, D. P. *et al.* The validity of two versions of the GHQ in the WHO study of mental illness in general health care. *Psychol. Med.* **27**, 191–197 (1997).

11. Juhola, J. *et al.* Internal consistency and factor structure of Jenkins Sleep Scale: cross-sectional cohort study among 80 000 adults. *BMJ Open* **11**, e043276–e043276 (2021).

12. Robine, J. & Jagger, C. CREATING A COHERENT SET OF INDICATORS TO MONITOR HEALTH ACROSS EUROPE: THE EUROREVES 2 PROJECT. *The Gerontologist* **42**, 35–35 (2002).

13. Ilmarinen, J., Tuomi, K. & Klockars, M. Changes in the work ability of active employees over an 11-year period. *Scand. J. Work. Environ. Health* **23**, 49–57 (1997).

14. Tuomi, K., Ilmarinen, J., Martikainen, R., Aalto, L. & Klockars, M. Aging, work, life-style and work ability among Finnish municipal workers in 1981—1992. *Scand. J. Work. Environ. Health* **23**, 58–65 (1997).

15. Ahlstrom, L., Grimby-Ekman, A., Hagberg, M. & Dellve, L. The Work Ability Index and single-item Question: Associations with Sick Leave, Symptoms, and health-a Prospective Study of Women on long-term Sick Leave. *Scand. J. Work Environ. & Health* **36**, 404–412 (2010).

16. Halonen, J. I. *et al.* Green and blue areas as predictors of overweight and obesity in an 8-year follow-up study. *Obes. Silver Spring Md* **22**, 1910–1917 (2014).

17. Ervasti, J. *et al.* Sickness absence diagnoses among abstainers, low-risk drinkers and at-risk drinkers: consideration of the U-shaped association between alcohol use and sickness absence in four cohort studies. *Addict. Abingdon Engl.* **113**, 1633–1642 (2018).

18. Heikkilä, K. *et al.* Job strain and tobacco smoking: an individual-participant data meta-analysis of 166,130 adults in 15 European studies. *PloS One* **7**, e35463–e35463 (2012).

19. Ervasti, J. *et al.* Does increasing physical activity reduce the excess risk of work disability among overweight individuals? *Scand. J. Work. Environ. Health* **45**, 376–385 (2019).
