# Supplementary material for: Development of a multifactorial prediction model for commute mode choice in 10 983 Finnish public sector employees: a cross-sectional study
Source: BMJ Open. 2024 Oct 16;14(10):e080276. doi: 10.1136/bmjopen-2023-080276 (PMC11487787; doi:10.1136/bmjopen-2023-080276)
Supplement: Supplementary file 3 [file bmjopen-2023-080276.reviewer_comments.pdf]

PEER REVIEW HISTORY

BMJ Open publishes all reviews undertaken for accepted manuscripts. Reviewers are asked to complete a checklist review form and are provided with free text boxes to elaborate on their assessment. These free text comments are reproduced below.

ARTICLE DETAILS

Title (Provisional)

Development of a Multifactorial Prediction Model for Commute Mode Choice in 10,983 Finnish Public Sector Employees: A Cross-sectional Study

Authors

Makkonen, Anna; Gluschkoff , Kia; Airaksinen, Jaakko; Halonen, Jaana I.; Salo, Paula; Ervasti, Jenni

---

VERSION 1 - REVIEW

**Reviewer**	**1**
**Name**	**Novack, Lena**
**Affiliation**	**Soroka University Medical Center**
**Date**	**04-Dec-2023**
**COI**	**None**

The manuscript explores the risk factors for sustainable modes of commuting to work. The authors reach out to a large number of responders, choosing out of them close to 11,000 employees. The response rate of 72% was also impressive.

Overall, the manuscript describes a diligently performed study that enabled the researchers to make valid conclusions. Using Lasso regression approach is another advantage, which helps the authors to come up with a relatively concise prediction model.

My comments are only minor. Please see them below, not in order of their importance.

(1) The manuscript needs to be proofread for typos and more. Here are some, but not all, examples:

• “length” instead of “length” in table 2

• Page 16, lines 15-17: “may not BE perceived as” instead of “may not perceived as”

• Page 3, Line 31: “Due to the interpretability of the results, the analyses were …” need to be revised for English

(2) How many missing values were there? Were models on “all available cases” used in addition to multiple imputations techniques? MI is usually not the first and only

methodology to be used, unless a lot of information is missing, hopefully MCAR. Hence, the manuscript should include a description of the extent of this missing data.

(3) Page 18, lines 10-19: these are not strictly conclusions from the study

(4) Isn't "1) daily driving (yes, no) equivalent to the negative of 2) daily cycling or walking to work (yes, no)." Or were there categories in the middle, e.g. partial walking/cycling and driving? It seems so, but I suggest writing it down explicitly

(5) The motivation of the study seems to inform an effective intervention, based on "…A survey that could be used to identify individuals likely to commute in a particular way could help when designing effective interventions that encourage switching to a more sustainable and healthier commute mode." However, the findings on sex, number of children 7-18, length of commute, BMI, physical activity can hardly be used for designing interventions. "The good work climate" is the only one that is theoretically modifiable.

I think the main take from the study findings is that the resultant model may allow policymakers to plan the commute. Based on the study model, one may predict the number of employees expected to walk/bike, which may justify certain urban planning interventions in developing biking routes and more. I recommend that the authors reconsider and perhaps realign their original intention in conducting this study.

**Reviewer**	**2**
**Name**	**Larouche, Richard**
**Affiliation**	**University of Lethbridge**
**Date**	**16-Mar-2024**
**COI**	**I have no competing interest in relation to this manuscript.**

This manuscript examines the factors associated with travel mode to work in a large sample of Finnish employees in the public sector. The manuscript fits within the journal aims and scope. I appreciate that the authors examined multiple psychosocial aspects of the workplace as predictors of travel mode. However, I have several concerns with the manuscript in its current form as stated below. I have not used the lasso procedure before, so I cannot comment on this specific aspect.

- Abstract: The first sentence of the conclusion should be revised substantially because you did not report reliability and because some of the correlates of driving vs. active travel are different, you would need more than 5 predictors. I would also remove "nudges" as it is odd that this is mentioned in the abstract, but not in the paper.
- Participants: Please add a short sentence about how participants' consent was obtained.

- Page 7: It is indicated that data were collected in 2020. It would be important to specify when in 2020 and to discuss how COVID-19 may have influenced travel behaviour. This should also be acknowledged and discussed as a limitation (unless data were collected before the outbreak of COVID-19).
- Page 7: Limiting the analysis to travel mode in the summertime is an unneeded study limitation. Instead, the authors could control for season in the models or stratify their results by season. I would argue that, if we are to be successful in increasing walking and cycling to improve health and reduce carbon emissions (as discussed by the authors), we should pay even more attention about how to promote these behaviours in less favourable seasons.
- Page 7: it is mentioned that "missing predictor data were imputed using single imputation". It is essential to indicate more specifically and justify which method was used to impute data. Multiple imputation is normally preferred over single imputation. The authors should also indicate (at least in supplementary files) the proportion of missing data for the variables that were imputed and the variables that were used to predict the missing data.
- Page 8: Is any information about the reliability and validity of the measure used to assess commuting mode available? If yes, this should be indicated in the manuscript. If not, this should be acknowledged as an important limitation.
- Measures: the authors provided no information about the psychometric properties of the variables assessed as potential predictors of travel mode. Such information should be added to the manuscript. If such information is lacking, this needs to be acknowledged as a limitation. At the very least, authors could report Cronbach's alpha for the scales employed; information on test-retest reliability and validity would be better.
- Page 9: Please provide more details about how physical activity was assessed. For example, which questionnaire was used and how were participants' responses converted to MET hours?
- Page 9: With the sample size that you have (especially if you remove the unnecessary exclusion based on season), I encourage you to examine the factors associated with walking and cycling separately. This would make your results more insightful to inform future interventions.
- Page 9: Prediction error depends on the quality of the data and relevance of predictors, so I would tone down the statement on lines ~52-54.
- Table 2: As presented, the table does not stand alone. Because you mentioned standardization on page 10, I guess that the odds ratios for continuous variables represent the effect of each standard deviation increase, but I'm not sure. This should be explained in the table footnote. For binary/categorical variables, please indicate in the reference group in each row.
- Was body mass index measured or self-reported? If it was self-reported, this needs to be acknowledged as a limitation.
- In general, I find that the discussion lacks substance. Many times, study findings are compared to only one or two previous studies. There is a lot of speculation about

variables not assessed in the study. Instead, I would focus more on the variables that you assessed and how your findings are consistent (or not) with previous literature (and, when available, systematic reviews).

- Page 16: For the paragraph discussing the finding that having school-aged children was associated with higher odds of driving, the authors should discuss about the findings of previous studies about parents' travel mode. For example, previous studies have found that a large proportion of parents drive their children to school on the way to work because they think it's faster and more convenient. Such evidence would be much more relevant to discuss here than weather or topography.
- Conclusion: The first three sentences of this paragraph are not conclusions. Instead, you could elaborate more on your findings and their implications for future research, policy, and/or practice.
- Grammar needs improvement. I have not taken that into account in my overall recommendation about the manuscript, but I would encourage the authors to use a language editing service.
- Through the manuscript, the concepts of sex and gender seem to be used interchangeably. Sex is a biological variable and gender is a social/cultural variable; they should not be used interchangeably. On page 8, it is important to specify how sex (or gender) was assessed.
- It is great that you shared code, but the data sharing statement should be about data sharing. Please review the author guidelines and update the statement accordingly.

**Reviewer**	**3**
**Name**	**Whyte, Bruce**
**Affiliation**	**University of Glasgow**
**Date**	**22-Mar-2024**
**COI**	**None**

Overall the paper is clearly written and the results are interesting and relevant to policy makers.

I cannot comment on the detail and robustness of the statistical analysis. A qualified statistician should be asked to review this part of the paper.

As noted by the authors, the study is limited by the fact that it is cross-sectional and can only show associations rather than causal links. While the authors explain their reasoning for grouping cyclist and pedestrian commuters together, this is a pity as there may have been associations specific to cyclist commuters and those specific to pedestrian commuters that are different.

The abstract states 'It is possible to reliably identify the determinants of active and passive commute choice….". This is over-stating the case. The factors identified are associated with the different modes of commuting but do not necessarily determine them. For example, that

an active commuter has a lower BMI and higher physical activity is strongly correlated with their choice to commute actively and arguably it is the active commuting that plays a large part in their being more active and having a lower BMI. A more cautious use of language (e.g. referring to 'associations' or 'correlations') would be more appropriate. In contrast we know from many studies that commute distance is likely to be a key determinant of choice of mode.

A 'good work climate' comes across as a strange expression. An alternatives might be a 'supportive or inclusive work environment' or just a 'positive work environment'. What constitutes this type of environment should be clearly explained. This is one of the most important correlates for policy as it is amenable to change and merits further explanation. The conclusions could be strengthened. More supportive work environments accompanied by suitable AT infrastructure can potentially encourage even more people to commute actively.

Some other minor points:

It would be useful to know how the final study population compares to the overall survey population and how representative it is of the Finish Public Sector overall. The Strengths and limitations section of the paper notes that the population may not be representative of the total working population of Finland. This point could be expanded upon in the discussion with specific quantifiable comparisons.

The paper would be benefit from proper proofing. There are quite a lot of sentences that have spelling mistakes or missing words e.g. page 3, line 45; page 13, lines 23 & 24 (Table 2); page 15, line 8; page 16, line 17. This is not a comprehensive list there are other typographical errors.

I do not understand the sentence or point being made on page 5 lines 45-46.

VERSION 1 - AUTHOR RESPONSE

Our responses can be found in the attached file below.

Reviewer 1
Dr. Lena Novack, Soroka University Medical Center, Ben-Gurion University of the Negev

Point 5: The manuscript explores the risk factors for sustainable modes of commuting to work. The authors reach out to a large number of responders, choosing out of them close to 11,000 employees. The response rate of 72% was also impressive.

Overall, the manuscript describes a diligently performed study that enabled the researchers to make valid conclusions. Using Lasso regression approach is another advantage, which helps the authors to come up with a relatively concise prediction model.

Author response: We thank the Reviewer for the positive feedback.

My comments are only minor. Please see them below, not in order of their importance.

Point 6: The manuscript needs to be proofread for typos and more. Here are some, but not all, examples:

- "length" instead of "length" in table 2
- Page 16, lines 15-17: "may not BE perceived as" instead of "may not perceived as"
- Page 3, Line 31: "Due to the interpretability of the results, the analyses were …" need to be revised for English

Author response: Thank you. We have corrected these and proofread the entire manuscript.

Point 7: How many missing values were there? Were models on "all available cases" used in addition to multiple imputations techniques? MI is usually not the first and only methodology to be used, unless a lot of information is missing, hopefully MCAR. Hence, the manuscript should include a description of the extent of this missing data.

Author response: We have described the extent of the missing data in Web Appendix Figure 1. (see the figure below). We have used a multivariate imputation technique with one imputed dataset instead of multiple datasets. As we understand it, the LASSO method doesn't work if multiple imputed datasets have been created. We used the PM and Logreg methods in the Mice package (multivariate imputation by chained equations) in R. We have now provided this information on page 8.

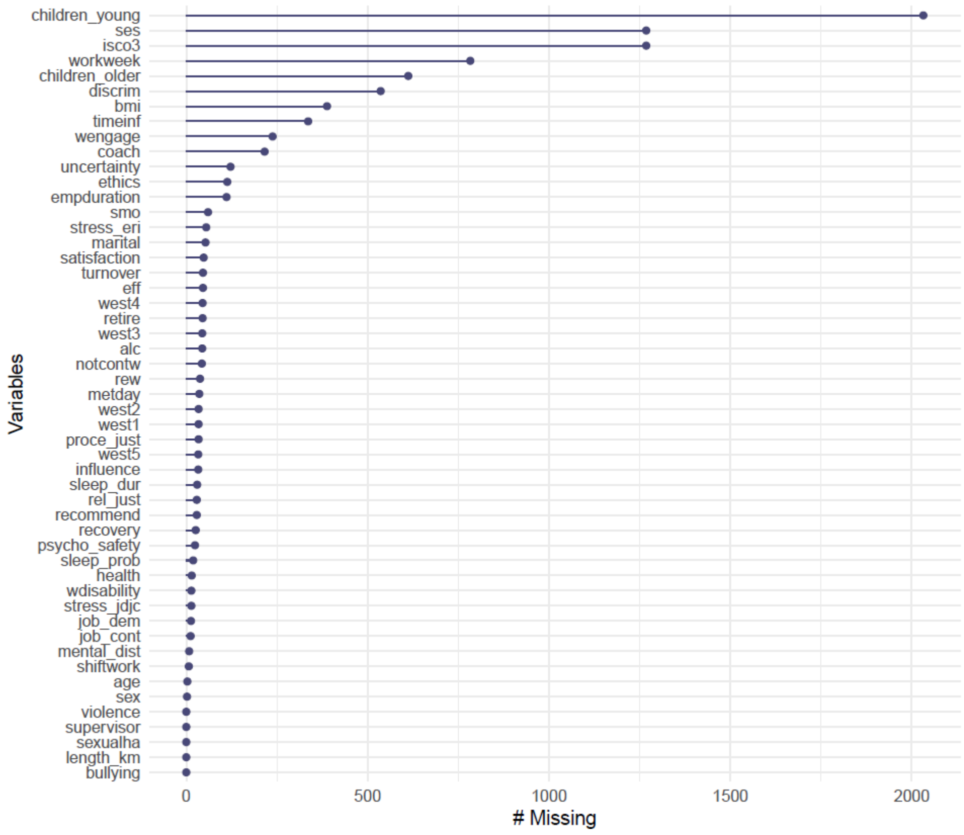

Web figure 1. The level of missing data before imputation

Point 8: Page 18, lines 10-19: these are not strictly conclusions from the study

Author response: We have now revised the conclusions to reflect more accurately our findings, as follows (p. 22-23):

"We created prediction models to identify the characteristics of individuals who are likely to choose a bike and those of a car commuter. Our prediction models, with eight predictors, were accurate at identifying the characteristics of these individuals who were active or passive commuters in summer and winter weather.

Alongside the better-known correlates, such as commute length and physical activity, we discovered a new, previously unidentified psychosocial factor associated with active commuting: psychological safety. Better team psychological safety was linked to daily active commuting during the summer weather. Notably, this factor can be modified with interventions. Workplaces with high psychological safety are likely also committed to the

overall well-being of their employees, including healthy work-life balance and promotion of physical activity. Psychological safety may be accompanied by empowerment and autonomy (Newman et al. 2017). Employees in control of their work and personal lives may be more inclined to take proactive measures to enhance their health and well-being, including opting for active commuting.

Our models provide a framework for intervention studies. If our findings can be replicated in other cohorts and are supported by intervention studies, policymakers, and urban planners can justify investments in biking routes and facilities, and employers in workplace environments that support active commuting, ultimately fostering healthier and more sustainable communities."

Newman, A., Donohue, R., & Eva, N. (2017). Psychological safety: A systematic review of the literature. *Human Resource Management Review*, *27*(3), 521–535. https://doi.org/10.1016/j.hrmr.2017.01.001

Point 9: Isn't "1) daily driving (yes, no) equivalent to the negative of 2) daily cycling or walking to work (yes, no)." Or were there categories in the middle, e.g. partial walking/cycling and driving? It seems so, but I suggest writing it down explicitly.

Author response: The question on commuting mode had multiple response options. We have clarified the response categories, as follows (p. 8-9):

"How often do you commute in the summer weather?" with response options (1) walking, (2) cycling, (3) public transport with 1000 m or more of walking or cycling, (4) public transport with less than 1000 m of walking or cycling, and (5) private car use, either as a driver or a passenger.

Point 10: The motivation of the study seems to inform an effective intervention, based on "…A survey that could be used to identify individuals likely to commute in a particular way could help when designing effective interventions that encourage switching to a more sustainable and healthier commute mode." However, the findings on sex, number of children 7-18, length of commute, BMI, physical activity can hardly be used for designing interventions. "The good work climate" is the only one that is theoretically modifiable.

Author response: Thank you, this is a very good point. We have now changed the conclusion accordingly (p. 22-23):

Alongside the better-known correlates, such as commute length and physical activity, we discovered a new, previously unidentified psychosocial factor associated with active commuting: psychological safety. Better team psychological safety was linked to daily active commuting during the summer weather. Notably, this factor can be modified with interventions. Workplaces with high psychological safety are likely also committed to the overall well-being of their employees, including healthy work-life balance and promotion of physical activity. Psychological safety may be accompanied by empowerment and autonomy (Newman et al. 2017). Employees in control of their work and personal lives may be more inclined to take proactive measures to enhance their health and well-being, including opting for active commuting.

Our models provide a framework for intervention studies. If our findings can be replicated in other cohorts and are supported by intervention studies, policymakers, and urban planners can justify investments in biking routes and facilities, and employers in workplace environments that support active commuting, ultimately fostering healthier and more sustainable communities."

Newman, A., Donohue, R., & Eva, N. (2017). Psychological safety: A systematic review of the literature. *Human Resource Management Review*, *27*(3), 521–535. https://doi.org/10.1016/j.hrmr.2017.01.001

Point 11: I think the main take from the study findings is that the resultant model may allow policymakers to plan the commute. Based on the study model, one may predict the number of employees expected to walk/bike, which may justify certain urban planning interventions in developing biking routes and more. I recommend that the authors reconsider and perhaps realign their original intention in conducting this study.

Author response: Thank you for the important notion. We have now realigned the study's intention as follows (page 2):

" To examine the feasibility of using survey data to identify factors that predict commute mode choice."

Reviewer: 2
Dr. Richard Larouche, University of Lethbridge

Point 12: This manuscript examines the factors associated with travel mode to work in a large sample of Finnish employees in the public sector. The manuscript fits within the journal aims and scope. I appreciate that the authors examined multiple psychosocial aspects of the workplace as predictors of travel mode. However, I have several concerns with the

manuscript in its current form as stated below. I have not used the lasso procedure before, so I cannot comment on this specific aspect.

Author response: Thank you for the positive feedback.

Point 13: Abstract: The first sentence of the conclusion should be revised substantially because you did not report reliability and because some of the correlates of driving vs. active travel are different, you would need more than 5 predictors. I would also remove "nudges" as it is odd that this is mentioned in the abstract, but not in the paper.

Author response: Thank you for the notion. We have revised the conclusion in the Abstract and the text in the conclusion section to more accurately reflect our findings. The sections now read as follows (p. 3):

"We identified the most important correlates of active and passive commute choice in different weather conditions with eight variables."

Point 14: Participants: Please add a short sentence about how participants' consent was obtained.

Author response: We have done as suggested (p.7-8):

"In the Finnish Public Sector (FPS) study, filling up the questionnaire is voluntary. The voluntariness is clearly stated in the cover letter of the questionnaire (information to study participants). We also inform the participants that filling out the questionnaire is considered consent to participate. Additionally, we ask informed consent to link survey data with register data."

Point 15: Page 7: It is indicated that data were collected in 2020. It would be important to specify when in 2020 and to discuss how COVID-19 may have influenced travel behaviour. This should also be acknowledged and discussed as a limitation (unless data were collected before the outbreak of COVID-19).

Author response: The data was collected in the fall of 2020, in September-October, and indeed, may have been influenced by COVID-19. We have now added the description of the time of data collection as follows (p. 7)

“The study population (N=58,971) included employees of four Finnish cities, with survey data collected from employees during September-October 2020.”

Moreover, we discuss this in the limitation section as follows (p. 22):

“Lastly, the data collection occurred in September-October 2020, after the outbreak of the COVID-19 pandemic. The pandemic may have led to more people commuting by car or active modes than normally.”

Point 16: Page 7: Limiting the analysis to travel mode in the summertime is an unneeded study limitation. Instead, the authors could control for season in the models or stratify their results by season. I would argue that, if we are to be successful in increasing walking and cycling to improve health and reduce carbon emissions (as discussed by the authors), we should pay even more attention about how to promote these behaviours in less favourable seasons.

Author response: We agree and have added results for both active and passive commuting also in winter weather (Table 3, Figure 2, Figure 4 and Web appendix Web table 3, Web table 4 Web figure 5, Web figure 6, Web figure 7 and Web figure 9).

Point 17: Page 7: it is mentioned that “missing predictor data were imputed using single imputation”. It is essential to indicate more specifically and justify which method was used to impute data. Multiple imputation is normally preferred over single imputation. The authors should also indicate (at least in supplementary files) the proportion of missing data for the variables that were imputed and the variables that were used to predict the missing data.

Author response: We used a multivariate imputation technique with one imputed dataset instead of multiple datasets. The LASSO method doesn't work if multiple imputed datasets have been created. We used the PM and Logreg methods in the Mice package (multivariate imputation by chained equations) in R. We describe the extent of the missing data in supplement Web Figure 1 and have revised the text accordingly, on page 8 as follows:

“We performed a multiple imputation with one imputed dataset using the R package Mice (multivariate imputation by chained equations) for the missing predictor data. The level of missing data before imputation is shown in Web figure 1. The final analytical sample consisted of 10,983 participants.”

Point 18: Page 8: Is any information about the reliability and validity of the measure used to assess commuting mode available? If yes, this should be indicated in the manuscript. If not, this should be acknowledged as an important limitation.

Author response: We have now clarified the commute mode measures (Point 9 above) and provide reference to previous studies using the same measure. Calculating reliability would require the measurement of a latent construct that cannot be measured directly. Instead, we measured commute modes directly. Regarding validity, we have previous articles (Kalliolahti et al., 2023; Haukka et al., 2023) published using this measure. The studies have observed relationships between commute mode, health, and workability. In other words, our measure of active commute has shown similar associations with health as observed in earlier studies, supporting its construct and face validity.

Kalliolahti, E., Aalto, V., Salo, P., Lanki, T., Ervasti, J., & Oksanen, T. (2023). Associations between commute mode use and self-rated health and work ability among Finnish public sector employees. Scandinavian Journal of Public Health, 14034948231159212–14034948231159212. https://doi.org/10.1177/14034948231159212

Haukka, E., Gluschkoff, K., Kalliolahti, E., Lanki, T., Jussila, J. J., Halonen, J. I., … Ervasti, J. (2023). Changes in active commuting and changes in health: Within- and between-individual analyses among 16 881 Finnish public sector employees. Preventive Medicine, 177, 107744–107744. https://doi.org/10.1016/j.ypmed.2023.107744

Point 19: Measures: the authors provided no information about the psychometric properties of the variables assessed as potential predictors of travel mode. Such information should be added to the manuscript. If such information is lacking, this needs to be acknowledged as a limitation. At the very least, authors could report Cronbach's alpha for the scales employed; information on test-retest reliability and validity would be better.

Author response: The psychometric properties of the explanatory variables have been assessed in the previous studies referenced in the article (e.g. Anderson et al. 1996; Goldberg et al.1997; Juhola et al. 2021; Kivimaki et al. 1999; Kivimäki et al. 2007; Robine et al. 2002). Some measures have not undergone psychometric testing; however, they have all been used in previous research. These earlier publications lend credibility to the measures, indicating they have performed as expected and their relationships with other variables are well-documented. We have now refined the web appendix where the measures are described in more detail.

Anderson, N. & West, M. A. The team climate inventory: Development of the tci and its applications in teambuilding for innovativeness. Eur. J. Work Organ. Psychol. 5, 53–66 (1996).

Goldberg, D. P. et al. The validity of two versions of the GHQ in the WHO study of mental illness in general health care. Psychol. Med. 27, 191–197 (1997).

Juhola, J. et al. Internal consistency and factor structure of Jenkins Sleep Scale: cross-sectional cohort study among 80 000 adults. BMJ Open 11, e043276–e043276 (2021).

Kivimaki, M. & Elovainio, M. A short version of the Team Climate Inventory: Development and psychometric properties. J. Occup. Organ. Psychol. 72, 241–246 (1999).

Kivimaki, M., Vahtera, J., Elovainio, M., Virtanen, M. & Siegrist, J. Effort-reward imbalance, procedural injustice and relational injustice as psychosocial predictors of health: complementary or redundant models? Occup. Environ. Med. 64, 659–665 (2007).

Robine, J. & Jagger, C. Creating a Coherent Set of Indicators to Monitor Health Across Europe: The Euroreves 2 Project. The Gerontologist 42, 35–35 (2002).

Point 20: Page 9: Please provide more details about how physical activity was assessed. For example, which questionnaire was used and how were participants' responses converted to MET hours?

Author response: We now provide more details about physical activity in the main file (p. 10) and in the web appendix (p. 6-7) as follows:

"Total physical activity was measured with questions on average weekly hours of physical activity or exercise during leisure time or commuting within the previous 12 months, with varying intensity corresponding to walking, brisk walking, jogging, and running. The response categories were: < 30 minutes, 1 hour, 2–3 hours, and > 4 hours. We used the following scales for calculations: <30 minutes = 15 minutes, 1 hour = 45 minutes, 2–3 hours = 2.5 hours, and >4 hours = 5 hours. The time spent on activity at each intensity level in hours per week was multiplied by the average energy expenditure of each activity and expressed in MET. Physical activities evaluated to correspond to walking, brisk walking, jogging, and running, were given MET values 3.5, 5, 8, and 11 MET, respectively. (Ervasti et al.2019)."

Ervasti J, Airaksinen J, Pentti J, et al. Does increasing physical activity reduce the excess risk of work disability among overweight individuals? Scandinavian Journal of Work, Environment & Health. 2019;45(4):376–385. https://doi.org/10.5271/sjweh.3799

Point 21: Page 9: With the sample size that you have (especially if you remove the unnecessary exclusion based on season), I encourage you to examine the factors associated

with walking and cycling separately. This would make your results more insightful to inform future interventions.

Author response: Thank you for this comment. The reason for combining walking and cycling into an active commute was that when the commute distance is more than 2 km, the number of people walking to work drops dramatically (Finnish Transport and Communications Agency Traficom 2023). In our sample, those walking to work regularly for more than two kilometers represented only 5.7 % of all respondents, and thus, we could not separately examine walking.

Finnish Transport and Communications Agency Traficom. Traficomin tutkimuksia ja selvityksiä 1/2023. Henkilöliikennetutkimus 2021 (traficom.fi).

Point 22: Page 9: Prediction error depends on the quality of the data and relevance of predictors, so I would tone down the statement on lines ~52-54.

Author response: We have now toned down the statement on page 11 as follows:

"The main advantage of the lasso model compared to more conventional regression models is its capability to minimize prediction error and to produce a parsimonious (or sparse) model."

Point 23: Table 2: As presented, the table does not stand alone. Because you mentioned standardization on page 10, I guess that the odds ratios for continuous variables represent the effect of each standard deviation increase, but I'm not sure. This should be explained in the table footnote. For binary/categorical variables, please indicate in the reference group in each row.

Author response: Thank you for this observation. We have now added the explanation in the footnote of Table 2. We have also indicated the reference groups for the categorical variables.

Point 24: Was body mass index measured or self-reported? If it was self-reported, this needs to be acknowledged as a limitation.

Author response: As our data were mostly from employee survey, practically all variables were self-reported, including body mass index. We now discuss this as a limitation, as follows (p. 22):

"All survey items were self-reported and therefore subject to recall and/or social desirability bias."

Point 25: In general, I find that the discussion lacks substance. Many times, study findings are compared to only one or two previous studies. There is a lot of speculation about variables not assessed in the study. Instead, I would focus more on the variables that you assessed and how your findings are consistent (or not) with previous literature (and, when available, systematic reviews).

Author response: Thank you for this notion. We added more references to previous studies and systematic reviews (introduction section, discussion, and limitations) and discussed our study's findings with previous literature.

New literature:

Bourne, J. E. *et al.* Health benefits of electrically-assisted cycling: a systematic review. *Int. J. Behav. Nutr. Phys. Act.* **15**, 116–116 (2018).

Chakrabarti, S. & Joh, K. The effect of parenthood on travel behavior: Evidence from the California Household Travel Survey. *Transp. Res. Part Policy Pract.* **120**, 101–115 (2019).

Chng, S., White, M., Abraham, C. & Skippon, S. Commuting and wellbeing in London: The roles of commute mode and local public transport connectivity. *Prev. Med.* **88**, 182–188 (2016).

Christiansen, P., Engebretsen, Ø., Fearnley, N. & Usterud Hanssen, J. Parking facilities and the built environment: Impacts on travel behaviour. *Transp. Res. Part Policy Pract.* **95**, 198–206 (2017).

Goel, R. *et al.* Cycling behaviour in 17 countries across 6 continents: levels of cycling, who cycles, for what purpose, and how far? *Transp. Rev.* **42**, 58–81 (2022).

Flint, E. & Cummins, S. Active commuting and obesity in mid-life: cross-sectional, observational evidence from UK Biobank. Lancet Diabetes Endocrinol. 4, 420–435 (2016).

Flint, E., Cummins, S. & Sacker, A. Associations between active commuting, body fat, and body mass index: population based, cross sectional study in the United Kingdom. BMJ Online 349, g4887–g4887 (2014).

de Haas, M., Kroesen, M., Chorus, C., Hoogendoorn-Lanser, S. & Hoogendoorn, S. Causal relations between body-mass index, self-rated health and active travel: An empirical study based on longitudinal data. *J. Transp. Health* **22**, 101113- (2021).

Haukka, E. *et al.* Changes in active commuting and changes in health: Within- and between-individual analyses among 16 881 Finnish public sector employees. *Prev. Med.* **177**, 107744–107744 (2023).

Henriques-Neto, D., Peralta, M., Garradas, S., Pelegrini, A., Pinto, A. A., Sánchez-Miguel, P. A., & Marques, A. (2020). Active commuting and physical fitness: A systematic review. *International Journal of Environmental Research and Public Health*, *17*(8), 2721-. https://doi.org/10.3390/ijerph17082721

Julsrud, T. E. & Hjorthol, R. Commuting in knowledge intensive organizations: An outline of six different practices. *Int. J. Sustain. Transp.* **15**, 943–957 (2021).

Khordagui, N. Parking prices and the decision to drive to work: Evidence from California. *Transp. Res. Part Policy Pract.* **130**, 479–495 (2019).

Kuwahara, K. et al. Association of changes in commute mode with body mass index and visceral adiposity: a longitudinal study. Int. J. Behav. Nutr. Phys. Act. 16, 101–101 (2019).

Newman, A., Donohue, R., & Eva, N. (2017). Psychological safety: A systematic review of the literature. Human Resource Management Review, 27(3), 521–535. https://doi.org/10.1016/j.hrmr.2017.01.001

Oakil, A. T. M., Manting, D. & Nijland, H. Dynamics in car ownership: the role of entry into parenthood. *Eur. J. Transp. Infrastruct. Res.* **16**, (2016).

Page, N. C. & Nilsson, V. O. Active Commuting: Workplace Health Promotion for Improved Employee Well-Being and Organizational Behavior. *Front. Psychol.* **7**, 1994–1994 (2017).

Rachele, J. N. *et al.* Associations between individual socioeconomic position, neighbourhood disadvantage and transport mode: baseline results from the HABITAT multilevel study. *J. Epidemiol. Community Health 1979* **69**, 1217–1223 (2015).

Rasca, S. & Saeed, N. Exploring the factors influencing the use of public transport by commuters living in networks of small cities and towns. *Travel Behav. Soc.* **28**, 249–263 (2022).

Soza-Parra, J. & Cats, O. The role of personal motives in determining car ownership and use: a literature review. *Transp. Rev.* **44**, 591–611 (2024).
Rasca, S. & Saeed, N. Exploring the factors influencing the use of public transport by commuters living in networks of small cities and towns. *Travel Behav. Soc.* **28**, 249–263 (2022).

Wojan, T. R. & Hamrick, K. S. Can Walking or Biking to Work Really Make a Difference? Compact Development, Observed Commuter Choice and Body Mass Index. *PloS One* **10**, e0130903–e0130903 (2015).

Point 26: Page 16: For the paragraph discussing the finding that having school-aged children was associated with higher odds of driving, the authors should discuss about the findings of previous studies about parents' travel mode. For example, previous studies have found that a large proportion of parents drive their children to school on the way to work because they think it's faster and more convenient. Such evidence would be much more relevant to discuss here than weather or topography.

Author response: We have revised this paragraph as follows (p. 19):

"Possible explanations exist for the association between living with school-aged children and driving. Time constraints, and greater necessity for trip chaining, such as dropping off or picking up children from schools or daycare centers, are direct effects of parenthood affecting commute mode choice (Chakrabarti et al. 2019; Motte-Baumvol. 2017). There are also

indirect effects, such as increased car ownership, that may significantly impact commute mode choice (Md Oakil et al. 2016; Sierra Muñoz et al. 2024).”

Chakrabarti, S., & Joh, K. (2019). The effect of parenthood on travel behavior: Evidence from the California Household Travel Survey. *Transportation Research. Part A, Policy and Practice*, *120*, 101–115. https://doi.org/10.1016/j.tra.2018.12.022

Md Oakil, A. T., Manting, D., & Nijland, H. (2016). Dynamics in car ownership: The role of entry into parenthood. *European Journal of Transport and Infrastructure Research*, *16*(4), 661–673. https://doi.org/10.18757/ejtir.2016.16.4.3164

Motte-Baumvol, B., Bonin, O., & Belton-Chevallier, L. (2017). Who escort children: mum or dad? Exploring gender differences in escorting mobility among parisian dual-earner couples. *Transportation (Dordrecht)*, *44*(1), 139–157. https://doi.org/10.1007/s11116-015-9630-1

Sierra Muñoz, J., Duboz, L., Pucci, P., & Ciuffo, B. (2024). Why do we rely on cars? Car dependence assessment and dimensions from a systematic literature review. *European Transport Research Review*, *16*(1), 17–16. https://doi.org/10.1186/s12544-024-00639-z

Point 27: Conclusion: The first three sentences of this paragraph are not conclusions. Instead, you could elaborate more on your findings and their implications for future research, policy, and/or practice.

Author response: The conclusion has now been revised as follows (p. 22-23):

“We created prediction models to identify the characteristics of individuals who are likely to choose a bike and those of a car commuter. Our prediction models, with eight predictors, were accurate at identifying the characteristics of these individuals who were active or passive commuters in summer and winter weather.

Alongside the better-known correlates, such as commute length and physical activity, we discovered a new, previously unidentified psychosocial factor associated with active commuting: psychological safety. Better team psychological safety was linked to daily active commuting during the summer weather. Notably, this factor can be modified with interventions. Workplaces with high psychological safety are likely also committed to the overall well-being of their employees, including healthy work-life balance and promotion of physical activity. Psychological safety may be accompanied by empowerment and autonomy (Newman et al. 2017). Employees in control of their work and personal lives may be more inclined to take proactive measures to enhance their health and well-being, including opting for active commuting.

Our models provide a framework for intervention studies. If our findings can be replicated in other cohorts and are supported by intervention studies, policymakers, and urban planners can justify investments in biking routes and facilities, and employers in workplace environments that support active commuting, ultimately fostering healthier and more sustainable communities."

Newman, A., Donohue, R., & Eva, N. (2017). Psychological safety: A systematic review of the literature. *Human Resource Management Review*, *27*(3), 521–535. https://doi.org/10.1016/j.hrmr.2017.01.001

Point 28: Grammar needs improvement. I have not taken that into account in my overall recommendation about the manuscript, but I would encourage the authors to use a language editing service.

Author response: The whole manuscript has been proofread.

Point 29: Through the manuscript, the concepts of sex and gender seem to be used interchangeably. Sex is a biological variable and gender is a social/cultural variable; they should not be used interchangeably. On page 8, it is important to specify how sex (or gender) was assessed.

Author response: We have changed gender to sex throughout the manuscript. *Sex* (man, woman), was collected from the employers' registers and is based on the social security number assigned to each citizen. This information has been added to the Web appendix and manuscript (p. 9).

Point 30: It is great that you shared code, but the data sharing statement should be about data sharing. Please review the author guidelines and update the statement accordingly.

Author response: We have now updated the data-sharing statement.

Reviewer: 3
Dr. Bruce Whyte, University of Glasgow

Point 31: Overall the paper is clearly written and the results are interesting and relevant to policy makers. I cannot comment on the detail and robustness of the statistical analysis. A qualified statistician should be asked to review this part of the paper.

Author response: Thank you for the positive feedback.

Point 32: As noted by the authors, the study is limited by the fact that it is cross-sectional and can only show associations rather than causal links. While the authors explain their reasoning for grouping cyclist and pedestrian commuters together, this is a pity as there may have been associations specific to cyclist commuters and those specific to pedestrian commuters that are different.

Author response: The reason for grouping pedestrian commuters and cyclists together is that, in Finland, according to the official statistics, when the commute distance is more than 2 km, the number of people walking to work drops dramatically (Finnish Transport and Communications Agency Traficom, 2021). In our sample, those walking to work regularly for more than two kilometers represented only 5.7 % of all respondents, and thus, we could not separately examine walking.

Finnish Transport and Communications Agency Traficom. Traficomin tutkimuksia ja selvityksiä 1/2023. Henkilöliikennetutkimus 2021 (traficom.fi).

Point 33: The abstract states 'It is possible to reliably identify the determinants of active and passive commute choice….". This is over-stating the case. The factors identified are associated with the different modes of commuting but do not necessarily determine them. For example, that an active commuter has a lower BMI and higher physical activity is strongly correlated with their choice to commute actively and arguably it is the active commuting that plays a large part in their being more active and having a lower BMI. A more cautious use of language (e.g. referring to 'associations' or 'correlations') would be more appropriate. In contrast we know from many studies that commute distance is likely to be a key determinant of choice of mode.

Author response: We agree and have now revised the text throughout the manuscript accordingly. The abstract now reads:

"We identified the most important correlates of active and passive commute choice in different weather conditions with eight variables. This information can be used to develop and target interventions to promote sustainable and healthy commuting modes."

We have also included previous studies, both cross-sectional and longitudinal, that examine the relationship between BMI and active commuting in the discussion section. New references are:

Henriques-Neto, D., Peralta, M., Garradas, S., Pelegrini, A., Pinto, A. A., Sánchez-Miguel, P. A., & Marques, A. (2020). Active commuting and physical fitness: A systematic review. *International Journal of Environmental Research and Public Health*, *17*(8), 2721-. https://doi.org/10.3390/ijerph17082721

Flint, E. & Cummins, S. Active commuting and obesity in mid-life: cross-sectional, observational evidence from UK Biobank. Lancet Diabetes Endocrinol. 4, 420–435 (2016).

Flint, E., Cummins, S. & Sacker, A. Associations between active commuting, body fat, and body mass index: population based, cross sectional study in the United Kingdom. BMJ Online 349, g4887–g4887 (2014).

Kuwahara, K. et al. Association of changes in commute mode with body mass index and visceral adiposity: a longitudinal study. Int. J. Behav. Nutr. Phys. Act. 16, 101–101 (2019).

Wojan, T. R. & Hamrick, K. S. Can Walking or Biking to Work Really Make a Difference? Compact Development, Observed Commuter Choice and Body Mass Index. *PloS One* **10**, e0130903–e0130903 (2015).

Point 34: A ‘good work climate’ comes across as a strange expression. An alternatives might be a ‘supportive or inclusive work environment’ or just a ‘positive work environment’. What constitutes this type of environment should be clearly explained. This is one of the most important correlates for policy as it is amenable to change and merits further explanation. The conclusions could be strengthened. More supportive work environments accompanied by suitable AT infrastructure can potentially encourage even more people to commute actively.

Author response: This is a very good point. We now follow the description used previously (Ervasti et al., 2023) and describe this measure as ‘team psychological safety’, which better describes the concept. We have now described the items in Web Appendix and modified the conclusions so that the modifiability of this factor is noticed. Please also see our response to points 10, 18-19.

Some other minor points:

Point 35: It would be useful to know how the final study population compares to the overall survey population and how representative it is of the Finish Public Sector overall. The Strengths and limitations section of the paper notes that the population may not be representative of the total working population of Finland. This point could be expanded upon in the discussion with specific quantifiable comparisons.

Author response: We have now added a description of the representativeness of the study population in the main text, in the participant section, as follows (p. 7):

"The study population (N=58,971) included employees of four Finnish cities, with survey data collected from employees in September-October 2020. A total of 42,574 employees (72%) responded. The sample comprised approximately 10% of all public sector employees in Finland in 2020. The majority (84%) of participants were women, which aligns with the general sex distribution of Finnish public sector workers. In 2020, the most common occupations in the Finnish municipal sector were those related to healthcare, social services, and education, representing nearly 50% of all occupational groups."

Strengths and limitations section (p. 21) as follows:

"Our analytical sample (Table 1.) represented the whole sample well, in which the proportion of women was 78.9 %, and the mean age was 46 years, with a standard deviation of 11."

Point 36: The paper would be benefit from proper proofing. There are quite a lot of sentences that have spelling mistakes or missing words e.g. page 3, line 45; page 13, lines 23 & 24 (Table 2); page 15, line 8; page 16, line 17. This is not a comprehensive list there are other typographical errors.

Author response: Thank you for noting this. We have now corrected the indicated sentences and proofread the whole manuscript.

Point 37: I do not understand the sentence or point being made on page 5 lines 45-46.

Author response: We have now revised the point as follows (p. 3):

"We identified the most important correlates of active and passive commute choice in different weather conditions with eight variables. This information can be used to develop and target interventions to promote sustainable and healthy commuting modes."

VERSION 2 - REVIEW

**Reviewer**	**2**
**Name**	**Larouche, Richard**
**Affiliation**	**University of Lethbridge**

Date **22-Jul-2024**

COI **N/A**

Thank you for addressing the comments from the reviewers. I appreciate that the authors have added models for the winter season and I think that the manuscript is substantially improved overall, but there are still a few issues to address as described below.

1. Abstract: In the conclusion, please delete “the most important”. There are likely many important correlates that were not assessed in this study, which is understandable, but the wording should be toned down here.

2. Methods – participants: I would recommend adding a justification for using single imputation in the manuscript. The authors provided one in the response to reviewers.

3. Methods – measures: I would not refer to the outcome as being “the most reliable” as reliability has not been assessed.

4. Methods – measures: I would suggest adding a brief definition of worktime control. Is this the perceived amount of control that participants have?

5. Methods – measures: I would add “self-reported” before body mass index.

6. Table 1: the numbers for “supervisor position” add up to 100.4%, likely due to rounding.

7. Discussion – 1st paragraph: the predictive ability of the models is described as “strong”, but only one of four models achieved the 0.80 benchmark mentioned in the methods.

8. Conclusion: in the last paragraph, I would replace “provide a framework” by “can help inform”. Most of the significant correlates are not variables that can be targeted by interventions, with the exception of psychological safety.

Suggestion for statistical analyses: If 5.7% of participants who lived more than 2 km away from their workplace walked, I suppose that a greater percentage walked shorter distances, so the sample would include several hundreds of walkers, making it possible to analyze walking and cycling separately. Because the correlates of walking and cycling are likely to be different, this could better inform future interventions. I think this is a lost opportunity, but I respect the authors’ decision.

VERSION 2 - AUTHOR RESPONSE

Reviewer 2
Dr. Richard Larouche, University of Lethbridge

Comments to the Author:

Point 1: Thank you for addressing the comments from the reviewers. I appreciate that the authors have added models for the winter season and I think that the manuscript is substantially improved overall, but there are still a few issues to address as described below.

Author response: We thank the Reviewer for the positive feedback.

Point 2: Abstract: In the conclusion, please delete “the most important”. There are likely many important correlates that were not assessed in this study, which is understandable, but the wording should be toned down here.

Author response: Thank you for this notion. We have now deleted the text in the abstract´s conclusion accordingly.

Point 3: Methods – participants: I would recommend adding a justification for using single imputation in the manuscript. The authors provided one in the response to reviewers.

Author response: Thank you for this notion. We have now added a justification for using multivariate imputation with one dataset in the manuscript (p. 8).

“We used only one imputed dataset, because using multiple datasets with LASSO would result in multiple sets of predictors that would differ from each other and could not be readily pooled.”

For clarification, the “traditional” method for imputation involves generating multiple datasets, running analyses on all the generated datasets, and then combining the results into a single set. With LASSO, this does not work, as the imputed datasets will all provide slightly varying sets of predictors that cannot readily be pooled.

Point 4: Methods – measures: I would not refer to the outcome as being “the most reliable” as reliability has not been assessed.

Author response: Thank you for this comment. We have now changed the text and referred to the outcome as “the most accurate.”

Point 5: Methods – measures: I would suggest adding a brief definition of worktime control. Is this the perceived amount of control that participants have?

Author response: Yes, this is a perceived amount of worktime control. We have added a brief definition of worktime control in the manuscript (p. 10).

"*Worktime control* (7 items) was measured using a questionnaire in which the participants were asked to evaluate how much they could influence the following aspects of their working time: length, starting and ending times, breaks, and handling of private matters during the workday, scheduling of work shifts, vacations and paid days off, and the taking of unpaid leave."

The description of the measure can also be found in the Web appendix (p. 2).

Point 6: Methods – measures: I would add "self-reported" before body mass index.

Author response: We have now added "self-reported" before body mass index.

Point 7: Table 1: the numbers for "supervisor position" add up to 100.4%, likely due to rounding.

Author response: Thank you for your attention. This has indeed been due to rounding. We have now adjusted the numbers to an integer (e.g. 9.4% to 9%).

Point 8: Discussion – 1st paragraph: the predictive ability of the models is described as "strong", but only one of four models achieved the 0.80 benchmark mentioned in the methods.

Author response: Thank you for this observation. We have now revised the section to refer to the models' predictive accuracy as 'good,' which better reflects the overall situation.

Point 9: Conclusion: in the last paragraph, I would replace "provide a framework" by "can help inform". Most of the significant correlates are not variables that can be targeted by interventions, with the exception of psychological safety.

Author response: Thank you for this comment. The text has been changed now as suggested.

Point 10: Suggestion for statistical analyses: If 5.7% of participants who lived more than 2 km away from their workplace walked, I suppose that a greater percentage walked shorter distances, so the sample would include several hundreds of walkers, making it possible to analyze walking and cycling separately. Because the correlates of walking and cycling are likely to be different, this could better inform future interventions. I think this is a lost opportunity, but I respect the authors' decision.

Author response: We appreciate the Reviewer´s suggestion. We have added a sentence in the limitations section (p. 23) stating that:

"In the future, it might also be useful to separate walkers and cyclists in the analyses, as their correlates may differ."

VERSION 3 - REVIEW

**Reviewer**	**2**
**Name**	**Larouche, Richard**
**Affiliation**	**University of Lethbridge**
**Date**	**06-Sep-2024**
**COI**	**None**

Thank you for considering my comments from the last round. I have no further comments.
